# Conservation Tillage-Mediated Rhizosphere Microbial Community Remodeling Drives Soil Organic Carbon Accumulation and Nitrogen and Phosphorus Transformation in Farmland

**DOI:** 10.3390/microorganisms14051092

**Published:** 2026-05-12

**Authors:** Haogeng Zhao, Meijuan Cheng, Shuli Wei, Gongfu Shi, Jing Fang, Huimin Shi, Qingze Liu, Yan Qu, Weijing Zhang, Fang Luo, Yu Wang, Zhanyuan Lu, Dejian Zhang, Xiaoqing Zhao

**Affiliations:** 1 School of Life Science, Inner Mongolia University, Hohhot 010020, China; 22408004@mail.imu.edu.cn (H.Z.); lqingze1995@163.com (Q.L.); 2 Inner Mongolia Academy of Agricultural & Animal Husbandry Sciences, Key Laboratory of Black Soil Protection and Utilization, Inner Mongolia Key Laboratory of Degradation Farmland Ecological Restoration and Pollution Control, Hohhot 010031, China; wslweishuli@163.com (S.W.); y214575@126.com (G.S.); nmgnongda@163.com (H.S.); quyan162643@163.com (Y.Q.); lzhy2811@163.com (Z.L.); 3 College of Agronomy, Inner Mongolia Agricultural University, Hohhot 010018, China; 2568509075@emails.imau.edu.cn (M.C.); fangjing721@163.com (J.F.); z13722009476@163.com (W.Z.); 4 Arun Banner Agricultural Technology Promotion Center, Hulun Buir 162750, China; arqtfz@163.com (F.L.); 15049521971@163.com (Y.W.)

**Keywords:** tillage measures, straw returning amount, nutrient element cycle, microbial function

## Abstract

Conservation tillage has an influence on the cultivation and sustainable utilization of farmland. However, the microbial mechanism driving soil nutrient cycling in conservation tillage and its regulation pathway remain unclear. Based on a positioning experiment in black soil areas, this study systematically compared the effects of no-tillage (NT) and moldboard tillage (MT) combined with different straw returning amounts (straw non-returning, NS; straw half-returning, HS; straw full-returning, TS) on the composition of soil carbon (C), nitrogen (N) and phosphorus (P) and focused on the role of microbial community structure succession and functional changes in soil nutrient cycling. Microbial community remodeling driven by tillage measures was mainly regulated by C and N components. Bacterial modules 2 and 4 and fungal modules 1 and 2 were key for regulating the C, N and P cycle, of which 87 bacteria and 45 fungi taxa represented the core driving microorganisms. The total amount of no-tillage straw return reduced the formation and accumulation of labile organic carbon fractions by enriching yeast-like fungi and inhibiting the expression of complex organic matter decomposition genes. Tillage mainly promoted the accumulation of labile organic carbon fractions and nutrient release by regulating the bacterial community, while no-tillage straw returning promoted the accumulation of total organic carbon and organic nitrogen fixation by promoting the fungal community. This study revealed the biological pathway of conservation tillage that drives soil nutrient cycling by regulating key microbial communities. It also provides a microbiological basis for sustainable soil management in black soil areas.

## 1. Introduction

The differences in tillage methods and management measures in farmland ecosystems directly lead to large spatial heterogeneity in the content of nutrients such as carbon, nitrogen and phosphorus and their metabolites, which in turn affects the cycling process of these nutrients [1]. Maintaining the accumulation, transformation and dynamic balance of carbon, nitrogen and phosphorus in soil is conducive to improving the stability of farmland ecosystems [2,3]. Reasonable tillage measures can effectively increase the content of nutrients and promote their mineralization and decomposition and enhance microbial activity as well as improve the soil structure and nutrient recycling, thereby increasing crop yields [4,5]. No-tillage and moldboard tillage significantly affect the morphology and distribution characteristics of soil carbon, nitrogen and phosphorus through different tillage disturbances. Conservation tillage with no-tillage and straw mulching at the core can significantly reduce soil erosion and increase the content of organic carbon and other nutrients by reducing soil disturbances and increasing ground roughness, which has become an important technical measure for farmland fertility and sustainable utilization [4,6,7,8]. Tillage accelerates the decomposition of organic matter by increasing mechanical disturbances, resulting in long-term soil organic carbon (SOC) loss [9]. No-tillage and straw returning can significantly increase the content of total carbon, nitrogen and phosphorus and organic carbon; increase the nitrogen and phosphorus storage capacity in surface soil [10,11,12]; and improve the utilization efficiency of soil phosphorus by increasing the turnover rate of microbial biomass phosphorus [13]. Straw returning promotes the accumulation of soil organic carbon by directly inputting organic matter, and the coupled carbon and nitrogen cycle may increase the total nitrogen content but also increases the gaseous nitrogen loss [14,15].

Soil microbial communities can quickly respond to changes in tillage methods, and community structures and functional gene expression will change significantly with tillage disturbances, then affecting soil nutrient cycles by regulating microbial assimilation and dissimilatory metabolism [16]. There are significant differences in soil microbial community compositions under different tillage methods, especially between no-tillage and moldboard tillage [17]. The soil bacterial community is mainly regulated by tillage methods, while the fungal community responds more significantly to management types but is also affected by tillage methods [18]. Long-term no-tillage measures can significantly increase the abundance of anaerobic microbial functional genes in soil, and the gene copy number can reach two to four times that of moldboard tillage soil. Combined with organic amendments, this can further promote the proliferation of anaerobic bacterial communities [19]. It has also been demonstrated that no-tillage straw returning significantly increases the relative abundance of acid bacteria but decreases the relative abundance of actinomycetes by reducing soil disturbances [20]; enhances the activity of beneficial microorganisms; avoids the destruction of arbuscular mycorrhizal fragile hyphal networks; increases the proportion of fungi and bacteria; and promotes the circulation of soil nutrients [21,22]. Plowing destroys the cascade interaction of microorganisms and weakens the function of fungi [23]. However, most of the existing studies focus on a single nutrient cycling process and changes in microbial community structures; therefore, changes in soil microbial-mediated carbon, nitrogen and phosphorus transformation and cycling function characteristics under different tillage measures are still unclear [24,25].

In this study, focusing on the problem that the driving mechanism of soil nutrient cycling microorganisms and the regulation pathway in conservation tillage farmland are not clear, a typical black soil farmland was treated with tillage measures such as no-tillage and straw mulching, and the effects of conservation tillage on soil nutrients and rhizosphere microorganisms and their interaction were systematically studied. The objectives of this study are as follows: (1) to clarify the changes in soil carbon, nitrogen and phosphorus components under conservation tillage measures; (2) to analyze the remodeling patterns of soil microbial community structure and their functional changes under conservation tillage; (3) to reveal the microbial-driven mechanisms of carbon, nitrogen and phosphorus cycles in conservation tillage farmland soils, and the following two hypotheses were proposed: (1) conservation tillage changes the diversity and community composition of maize rhizosphere microorganisms, in which bacterial and fungal groups show different response patterns.; (2) rhizosphere microorganisms enriched under conservation tillage drive the cycling and transformation of soil carbon, nitrogen and phosphorus by regulating their own metabolic functions. The research results provide an important theoretical basis for soil fertility cultivation and productivity improvements in black soil areas.

## 2. Materials and Methods

### 2.1. Experimental Design and Soil Sampling

The field experiment was carried out in 2021 at the Arongqi Long-Term Observation and Experimental Station of Soil Management and Ecological Restoration of the Inner Mongolia Academy of Agricultural and Animal Husbandry Sciences in northeast China (47°56′–49°19′ N, 122°2′–124°5′ E), which belongs to the temperate continental monsoon climate with the soil type classified as Chernozems. The average annual temperature is 1.7 °C, the frost-free period is 90–130 d, the annual precipitation is 458.4 mm and the annual sunshine hours are 2750–2850 h. Corn (*Zea mays* L.) was used as the test crop, and the experiment adopted a split-plot design, with tillage method as the main-plot treatment and straw returning amount as the sub-plot treatment. Six experimental treatments were set up: straw non-returning tillage (NSMT), straw half-returning tillage (HSMT), straw full-returning tillage (TSMT), straw non-returning no-tillage (NSNT), straw half-crushing no-tillage (HSNT) and straw full-crushing no-tillage (TSNT). Each treatment was repeated three times for a total of 18 plots. Each plot interval was 1.5 m, with an area of 330 m^2^ and a corn planting density of 82,500 plants/ha. The straw was crushed and returned to the field after maize harvests, and the crushing length was 5–10 cm. The straw was not returned to the field in order to remove the surface straw using a manual method. The straw application rates for the half-amount returns and full-amount returns were 5 t hm^−2^ and 10 t hm^−2^, respectively. The straw in the no-tillage mode was crushed and covered on the surface according to its application amount. In addition to the use of a double-row no-tillage planter, no other soil disturbances were carried out. The plowing treatment utilized a plough to turn the crushed straw into a soil layer, according to the degree of application, mixed the upper and lower soil layers evenly and then raked the ground. The burial depth was 30 cm. All treatments received the same fertilization rate: compound fertilizer was applied at 450 kg hm^−2^ (N:P_2_O_5_:K_2_O = 19:19:19), and urea was top dressed at 125 kg hm^−2^ at the jointing stage of corn, and the field management was the same as the conventional management measures. At the beginning of the experiment in 2021, the soil pH was 8.0, the soil organic carbon (SOC) was 20.8 g kg^−1^, the soil total nitrogen (TN) was 1.8 g kg^−1^, the soil total phosphorus (TP) was 0.65 g kg^−1^, the soil available phosphorus (AP) was 5.12 mg kg^−1^, the soil available potassium (AK) was 30.2 mg kg^−1^, and the soil total potassium (TK) was 16.92 g kg^−1^; all indicators were measured following the general conventional methods [26,27,28,29]; the detailed analytical methods are provided in Supplementary Text S1.

The results of two consecutive years of observation in 2022 and 2023 showed that there were significant differences in soil nutrient content and plant phenotypic traits (Table S1). On this basis, rhizosphere soil samples were collected for microbial diversity and nutrient composition analysis at the tasseling and silking stage of maize in August 2024. Ten maize plants with uniform growth were selected in each experimental plot, and the roots were completely excavated. After removing the loose attached soil, the rhizosphere soil within the range of 0–1 mm of the root was collected using a sterile brush, and then fully mixed to form a sample. Subsequently, fresh soil samples were filtered using a 2 mm aperture sieve to remove visible impurities. One part of the sample was dried and used for physical and chemical analysis, the other part was stored in a refrigerator at 4 °C for measuring microbial biomass, and the last part was stored in a refrigerator at −80 °C for soil microbial sequencing. The soil physical and chemical properties measured in this study are divided into three categories: carbon component, nitrogen component and phosphorus component. The nitrogen component test indicators include total nitrogen (TN), ammonium nitrogen (AN), nitrate nitrogen (NN), microbial biomass nitrogen (MBN), alkali-hydrolysable nitrogen (AHN) and organic nitrogen component (No); carbon components include organic carbon (SOC), microbial biomass carbon (MBC), dissolved organic carbon (DOC), easily oxidizable organic carbon (EOC), particulate organic carbon (POC), light fraction organic carbon (LFOC) and heavy fraction organic carbon (HFOC). The phosphorus components include total phosphorus (TP), available phosphorus (AP), microbial biomass phosphorus (MBP), organic phosphorus group (Po) and inorganic phosphorus group (Pi). All indicators were measured following the general conventional methods [30,31,32]; the detection methods of each index are detailed in the Supplementary Material (Text S1).

### 2.2. DNA Extraction and PCR Amplification

Total DNA was extracted from 0.05 g rhizosphere soil samples using the FastDNA^®^ SPIN Soil Extraction Kit (MP Biomedicals, Santa Ana, CA, USA). Subsequently, amplicon sequencing was performed on all DNA samples. Amplifier sequencing of the V4–V5 region of the bacterial 16S rRNA gene and the fungal internal transcribed spacer (ITS1) was performed by Meiji Biotechnology Co., Ltd. (Shanghai, China) on the Illumina Hiseq PE2500 platform [33]. Bacterial 16 SrRNA gene amplification using primer set 515 F/806 R, fungal ITS1 gene amplification using ITS1 F/ITS2 R (Table S2), in Text S2 provides more details about high-throughput sequencing. A total of 12,060 bacterial ASVs and 2481 fungal ASVs were obtained according to the minimum sample sequence depth.

### 2.3. Network Analysis

In order to explore the interaction pattern of rhizosphere microbial communities, the co-occurrence networks of bacteria and fungi were constructed based on the microbial data of all rhizosphere soil samples [34]. During the construction process, ASVs with an average relative abundance greater than 0.5% were used as network nodes. The Spearman rank correlation coefficient between nodes was calculated using the ‘psych’ package, and the nodes and edges with |r| > 0.70 and *p* < 0.01 (corrected by FDR) were screened to form a preliminary correlation network [35,36]. Network construction and visualization were completed by ‘igraph’ package and Gephi 0.0.1 software, respectively [37]. Furthermore, the ‘subgraph’ function of the ‘igraph’ package was used to extract the sub-networks of each treatment group from the overall network and calculate its topological parameters [38,39]. Subsequently, principal coordinate analysis (PCoA) was performed based on the topological characteristics of the sub-network to obtain indicators reflecting the complexity of the microbial network. In addition, the fast greedy algorithm was used to divide the network modules at the genus level. The minimum module size was set to 20 nodes, and the structural stability was enhanced by merging smaller modules. The modular results were visualized and analyzed in Cytoscape (version 3.10.4).

### 2.4. Microbial Function Analysis and Enzyme Activity Determination

The PICRUSt2 software(version 2.6.0) was used to analyze the high-throughput sequencing data of bacterial and fungal communities. Based on the KEGG (Kyoto Encyclopedia of Genes and Genomes) database, the abundance information of enzyme functional genes was obtained. The key enzyme genes related to carbon, nitrogen and phosphorus cycle were screened out. A total of 19 carbon cycle-related enzyme genes, 13 nitrogen cycle-related enzyme genes and 9 phosphorus cycle-related enzyme genes (Table S3) were identified in the bacterial community. The functional prediction results of the fungal community identified 12 carbon cycle-related enzyme genes, 6 nitrogen cycle-related enzyme genes and 8 phosphorus cycle-related enzyme genes (Table S4). The ‘igraph’ package was used to construct an interaction network containing key microbial groups, enzyme functional genes and soil carbon, nitrogen and phosphorus components. To ensure the capture of biologically meaningful weak correlations while avoiding excessive network fragmentation, a relatively loose screening threshold (|r| > 0.60 and *p* < 0.05) was adopted to retain nodes and edges [40,41]. A complete microorganism–enzyme gene-nutrient pathway requires that two conditions be met simultaneously: the microorganism is significantly correlated with the enzyme gene, and the enzyme gene is significantly correlated with the nutrient component. The network was visualized using Cytoscape (Version 3.10.4). The study revealed the potential path of key microorganisms to drive soil nutrient cycling by regulating specific enzyme genes.

In order to characterize the intensity of the soil nutrient cycling process under different tillage measures, the activities of soil urease, hydroxylamine reductase, β-glucosidase, β-xylosidase, polyphenol oxidase and peroxidase were further determined. The specific determination methods of each index are shown in Supplementary Materials (Text S3).

### 2.5. Statistical Analysis

All statistical analysis and chart visualization were performed in R (version 4.4.1). Two-way ANOVA was used to evaluate the effects of tillage methods, straw returning amount and their interactions on soil nutrients and microbial characteristics. Pearson correlation analysis was used to clarify the correlation between soil nutrient elements and microbial communities. Based on the R language ‘vegan’ package, principal coordinate analysis (PCoA) and permutation multivariate analysis of variance (ADONIS) were used to quantitatively analyze the variation characteristics of soil nutrient elements between different treatments. Through the PERMANOVA analysis of 999 replacements, the contribution rate of tillage methods and straw returning to the nutrient cycle process was further clarified. Variance decomposition analysis (VPA) based on redundancy analysis, Mantel test and random forest model analysis were performed using the R software package ‘randomForest’ and ‘vegan’ packages to explore the contribution of soil carbon, nitrogen and phosphorus components to the succession of microbial communities and to screen the core microorganisms that affect the nutrient cycle [42]. The partial least squares path model (PLS-PM) based on the ‘plspm’ package was used to evaluate the correlation between tillage practices, microbial communities and nutrient cycling, and the bootstrap method (1000 iterations) was used to verify the estimated values of the path coefficient and the coefficient of determination (R^2^). Finally, the overall prediction performance of the model was evaluated by the goodness-of-fit index (GFI) [43].

## 3. Results

### 3.1. Changes in Soil Carbon, Nitrogen and Phosphorus Fractions Under Conservation Tillage

**Figure 1:**
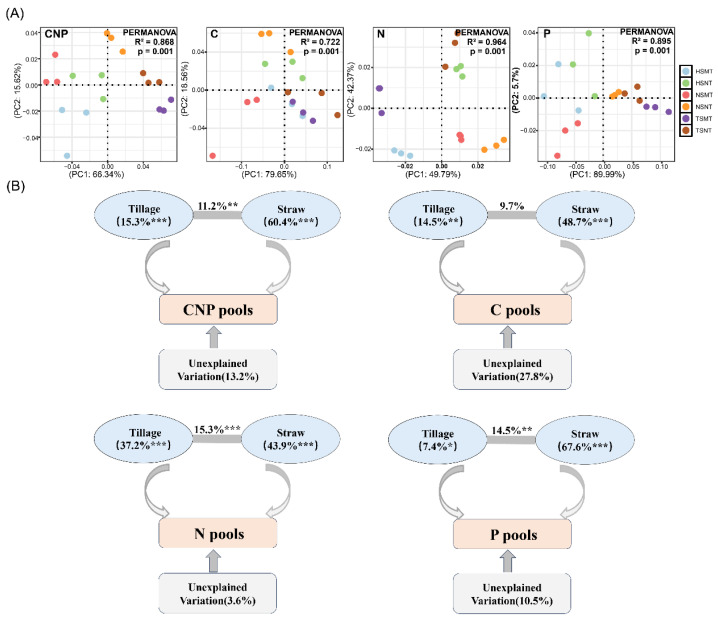
Principal coordinate analysis of soil carbon, nitrogen and phosphorus component contents under different treatments (**A**) and variance partitioning of the effects of straw return amount and tillage practices on soil carbon, nitrogen and phosphorus components based on PERMANOVA (**B**). Significant results are indicated by * *p* < 0.05, ** *p* < 0.01, *** *p* < 0.001.

The tillage methods and straw returning amount had significant regulatory effects on carbon, nitrogen and phosphorus components in maize rhizosphere soil (*p* < 0.05). The contents of soil organic carbon (SOC) and microbial biomass carbon (MBC) in the HSNT and TSNT treatments were significantly higher than those in the NSMT treatment, with increases of 20.17%, 14.52% (SOC), 42.11% and 78.68% (MBC) (*p* < 0.05). In general, the contents of the soil dissolved organic carbon (DOC), particulate organic carbon (POC) and light fraction organic carbon (LFOC) in the no-tillage treatment (NT) were lower than those in the tillage treatment (MT). With the increase in the straw returning amount, the amount of SOC, DOC and MBC increased, while the contents of POC and easily oxidized organic carbon (EOC) decreased (Figure S1). In terms of nitrogen components, compared with the NSMT treatment, the no-tillage straw returning (HSNT and TSNT) significantly increased the soil total nitrogen (TN), microbial biomass nitrogen (MBN), alkali-hydrolyzed nitrogen (AHN) and amino sugar nitrogen (ASN) content (*p* < 0.05). The tillage treatment exhibited higher available nitrogen (AN, NN) contents (*p* < 0.05; Figure S2). For phosphorus fractions, tillage methods were the main factors affecting the soil available phosphorus (AP) and total inorganic phosphorus (TPi) (*p* < 0.05) content, and the amount of straw returning significantly affected soil total phosphorus (TP), AP, total organic phosphorus (TPo) and TPi contents (*p* < 0.05). The tillage treatment (MT) significantly increased the AP content compared with the no-tillage treatment (NT). There were significant differences in the phosphorus content between different straw returning treatments, and the phosphorus content of the TSMT treatment was significantly higher than that of other treatments (*p* < 0.05; Figure S3). The results of the principal coordinate analysis (PCoA) demonstrated that there were significant differences in the overall and individual contents of soil carbon, nitrogen and phosphorus components across different treatments (*p* < 0.01; Figure 1A). A further analysis using PERMANOVA revealed that the amount of straw returning was the main factor that affected the variation of the carbon, nitrogen and phosphorus components in the rhizosphere of maize, and the tillage method also had a significant effect on the variation in these components (*p* < 0.05; Figure 1B).

### 3.2. Soil Microbial Community Remodeling Under Conservation Tillage Treatment

**Figure 2:**
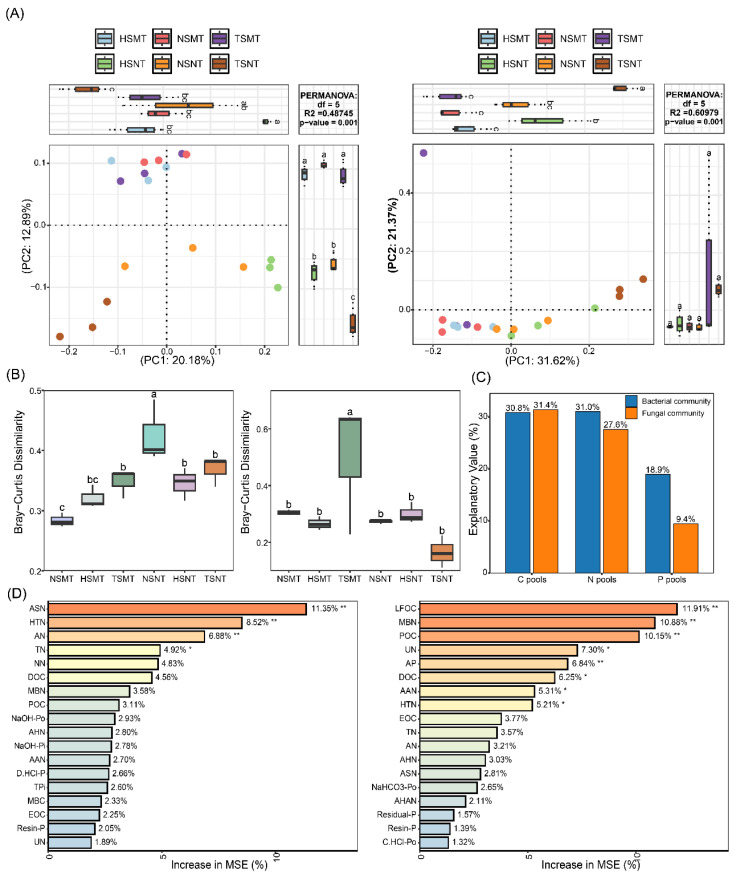
Principal component analysis (PCA) of soil bacterial and fungal communities under different tillage practices (**A**); Boxplots showing Bray–Curtis dissimilarities of bacterial and fungal communities among different tillage treatments (**B**). Different letters above the boxes indicate significant differences (*p* < 0.05) between treatments; variation partitioning analysis based on redundancy analysis of bacterial and fungal communities (**C**). The C pool consists of SOC, DOC, EOC, POC, HFOC, LFOC and MBC; the N pool consists of TN, NN, AN, AHN, HTN, AAN, ASN, AHAN, UN and MBN; the P pool contains TP, AP, MBP, Resin-P, NaHCO_3_-Pi, NaOH-Pi, D.HCl-Pi, C.HCl-Pi, NaHCO_3_-Po, NaOH-Po, C.HCl-Po and Residual-P. Random forest analysis showing the importance ranking of factors influencing bacterial and fungal communities, Variable importance was assessed by the increase in mean squared error (MSE, %) upon permutation of each variable. Higher values indicate greater importance of the variable in predicting microbial community composition (**D**). NSMT: moldboard tillage without straw incorporation; HSMT: moldboard tillage with half straw mulching; TSMT: moldboard tillage with full straw mulching; NSNT: no-tillage without straw incorporation; HSNT: no-tillage with half straw incorporation; TSNT: no-tillage with full straw incorporation. Significant results are indicated by * *p* < 0.05, ** *p* < 0.01.

A microbial alpha diversity analysis based on the Shannon and Chao1 indices demonstrated that different tillage methods significantly affected the soil bacterial community structure (*p* < 0.05). Compared with the tillage treatment (MT), the no-tillage treatment (NT) significantly reduced the bacterial Chao1 index and Shannon index (*p* < 0.05; Figure S4). The analysis of microbial community structures demonstrated that Pseudomonadota, Actinomycetota and Acidobacteriota were the dominant bacteria. The TSNT treatment significantly increased the relative abundance of Pseudomonadota and Actinomycetota (*p* < 0.05), while the HSNT treatment caused the highest level of Acidobacteriota’s relative abundance. The fungal community was dominated by Ascomycota and Basidiomycota, which together accounted for more than 80% of the relative abundance. The TSNT treatment significantly reduced the relative abundance of Ascomycota (*p* < 0.05) and significantly increased the abundance of Basidiomycota (*p* < 0.05; Figure S5). The results of the RF analysis of the microbial community and soil carbon, nitrogen and phosphorus components revealed that the relative abundance of Pseudomonadota and Actinomycetota was significantly positively correlated with soil organic carbon, total phosphorus and available nitrogen contents (*p* < 0.05) and significantly negatively correlated with organic nitrogen components (*p* < 0.05), but the abundance of other bacterial groups was significantly positively correlated with the organic nitrogen content (*p* < 0.05). In the fungal community, with the increase in the soil phosphorus content, the relative abundance of most fungal phyla decreased significantly (*p* < 0.05). In addition, the soil organic carbon content was negatively correlated with the Basidiomycota abundance but was positively correlated with other fungal groups (*p* < 0.05; Figure S6). The results of the PCOA further demonstrated that there were significant differences in bacterial and fungal community structures across different tillage methods (*p* < 0.01; Figure 2A). The results of the β-diversity analysis demonstrated that bacterial β-diversity was the greatest in the NSNT treatment, and the TSMT treatment significantly increased the fungal β-diversity (*p* < 0.05; Figure 2B). Results of the variance partitioning analysis (VPA) demonstrated that soil carbon and nitrogen components explained 62.2% and 59.0% of the bacterial and fungal community changes, respectively, while soil phosphorus components only explained 18.9% and 9.4% of the bacterial and fungal community changes, respectively (Figure 2C). The random forest model (Figure 2D) and Mantel test analysis (Figure S7) further confirmed that changes in the composition of bacterial and fungal communities were significantly correlated primarily with carbon and nitrogen components, including ASN, HTN, DOC, UN and MBN (*p* < 0.05).

### 3.3. The Structure and Function Differentiation of Soil Microbial Networks Under the Influence of Conservation Tillage

**Figure 3:**
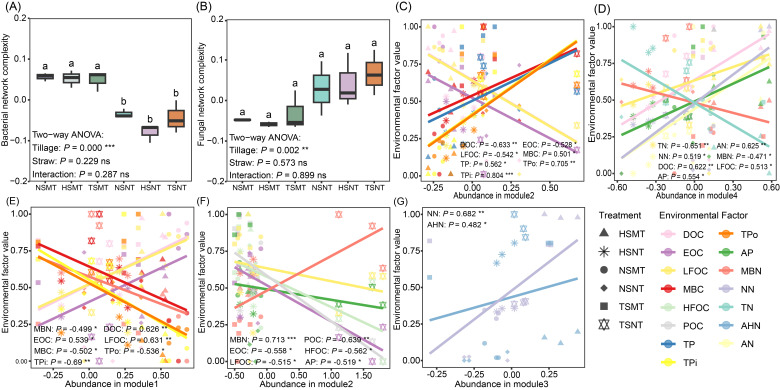
Bacterial (**A**) and fungal (**B**) network complexity based on principal component extraction; (**C**,**D**) correlation between bacterial module abundance and ecosystem function; (**E**–**G**) correlation between fungal module abundance and ecosystem function. Different lowercase letters indicate significant differences between treatments (*p* < 0.05); *, **, and *** represent treatment effects reaching significance levels of *p* < 0.05, *p* < 0.01, and *p* < 0.001, respectively, while NS denotes no significant effect. NSMT: moldboard tillage without straw incorporation; HSMT: moldboard tillage with half straw mulching; TSMT: moldboard tillage with full straw mulching; NSNT: no-tillage without straw incorporation; HSNT: no-tillage with half straw incorporation; TSNT: no-tillage with full straw incorporation.

The study constructed microbial networks of bacteria, fungi and under different treatments at the ASV level to compare the network complexity among different treatments (Figure S8). The results showed that the topological parameters of the bacterial and fungal networks only exhibited significant differences among different farming methods (*p* < 0.05). In the bacterial network, the number of nodes and edges, the average degree and the proportion of positive edges were significantly higher under moldboard tillage (MT) than in no-tillage (NT) treatments (Figure S9). In the fungal network, the number of edges, average degree, proportion of positive edges and network density were significantly higher under MT than in NT (*p* < 0.05), whereas the average path length and betweenness centrality were higher under NT (*p* < 0.05; Figure S10). A correlation analysis comparing microbial network topological parameters and soil carbon, nitrogen and phosphorus components revealed that the soil available nitrogen, carbon components and available phosphorus (AP) content were significantly positively correlated with most topological parameters (*p* < 0.05), while total nitrogen (TN) and organic nitrogen components were significantly negatively correlated with multiple topological parameters (*p* < 0.05; Figure S11). Based on the principal component extraction, the network complexity results demonstrated that the MT treatment significantly increased the complexity of the bacterial network (*p* < 0.05; Figure 3A,B).

Microbial networks were modularized at the genus level, with the bacterial and fungal networks partitioned into four and three major modules, respectively (Figure S12). To explore the potential ecological functions of each module, a linear regression analysis was conducted to examine the relationships between module relative abundance and soil carbon, nitrogen and phosphorus nutrient contents. This study found that bacterial modules 2 and 4 were essential for regulating soil nutrient cycling. Bacterial module 2 demonstrated a significant negative correlation with soil labile organic carbon components and significant positive correlations with total phosphorus (TP), organic phosphorus (TPo) and inorganic phosphorus (TPi) contents (*p* < 0.05). Bacterial module 4 was negatively correlated with total nitrogen (TN) and microbial biomass nitrogen (MBN) but positively correlated with available nitrogen (AN and NN), dissolved organic carbon (DOC) and light fraction organic carbon (LFOC), while it exhibited a negative correlation with available phosphorus (AP) (*p* < 0.05; Figure 3C,D). The ecological functions of the fungal community were primarily driven by modules 1 and 2, both of which significantly influenced soil labile organic carbon, organic/inorganic phosphorus and microbial biomass nitrogen contents. In contrast, module 3 was only significantly positively correlated with soil nitrate nitrogen (NN) and alkali-hydrolyzable nitrogen (AHN) contents (*p* < 0.05; Figure 3E–G).

An analysis of the microbial composition within the modules revealed that key microbial taxa influencing carbon, nitrogen and phosphorus cycling exist among the dominant microbial groups in each module. Specifically, bacterial module 2 contained microorganisms associated with ammonia oxidation, nitrogen fixation and organic carbon mineralization, such as *Nitrososphaeraceae*, *Candidatus_Nitrosocosmicus*, *Xanthobacteraceae* and *Gaiellales*. Bacterial module 4 was enriched with various taxa related to carbon cycling, including *Sphingomonas*, *Candidatus_Udaeobacter*, *Vicinamibacterales*, *Vicinamibacteraceae* and *Terriglobales*. In fungal module 1, the dominant taxa included *Poaceascoma*, *Periconia* and *Pleosporales*, which are involved in carbon decomposition. Fungal module 2 was primarily dominated by carbon-cycling-related microorganisms but also included taxa involved in phosphorus cycling, such as *Penicillium* and *Aspergillus*. In contrast, fungal module 3 contained multiple microorganisms capable of synergistically regulating carbon, nitrogen and phosphorus nutrient cycling, such as *Mortierella* and *Pseudogymnoascus* (Figure S13). To identify key microbial taxa regulating nutrient cycling within each module, a random forest model was applied to analyze the relative abundance of microorganisms. The results identified 87 and 45 potential key microbial taxa in the bacterial (Figure S14) and fungal (Figure S15) modules, respectively. These microorganisms were defined as core taxa driving differences in nutrient cycling across different treatments.

### 3.4. The Pathway of Core Soil Microbial Regulatory Functional Genes Affecting Nutrient Cycling

**Figure 4:**
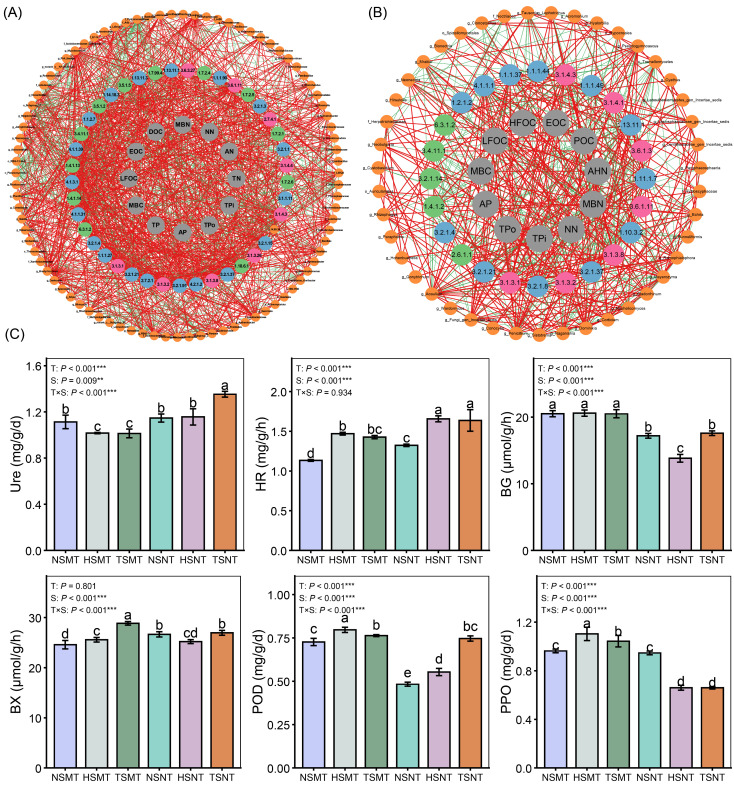
The functional network of soil core microorganisms in regulating nutrient cycling and the changes in key soil enzyme activities. Co-occurrence networks of core microorganisms regulating enzyme functional genes for bacteria (**A**) and fungi (**B**); (**C**) changes in soil enzyme activities related to carbon and nitrogen cycling under different treatments. The abundances of core microorganisms, carbon cycling enzyme functional genes, nitrogen cycling enzyme functional genes, phosphorus cycling functional genes and soil nutrient content are represented by dark yellow, blue, pink, green and gray, respectively. Urease (Ure), hydroxylamine reductase (H), β-glucosidase (BG), β-xylosidase (BX), polyphenol oxidase (PPO) and peroxidase (POD). Different lowercase letters indicate significant differences between treatments (*p* < 0.05); Significance level markers: ** *p* < 0.01, *** *p* < 0.001. NSMT: moldboard tillage without straw incorporation; HSMT: moldboard tillage with half straw mulching; TSMT: moldboard tillage with full straw mulching; NSNT: no-tillage without straw incorporation; HSNT: no-tillage with half straw incorporation; TSNT: no-tillage with full straw incorporation.

In this study, multiple functional enzyme genes associated with key biogeochemical cycles were identified based on the PICRUSt2 platform. By constructing relationship networks between core microorganisms; enzyme genes; and carbon, nitrogen and phosphorus components (Figure 4A,B), potential pathways by which microorganisms drive nutrient cycling by regulating specific enzyme genes were revealed (Figure S16). This study found that one bacterial and seven fungal microorganisms could participate in soil carbon and nitrogen cycling by regulating the expression of related functional enzyme genes. Among them, Actinomycetota significantly reduced soil microbial biomass nitrogen (MBN) levels by inhibiting the expression of the urease gene. In contrast, soil carbon components were primarily regulated by fungal microorganisms. *Echria* negatively regulated the expression of the formate dehydrogenase gene, leading to a decrease in the easily oxidizable organic carbon (EOC) content. Meanwhile, *Tausonia* and *Naganishia* reduced the soil particulate organic carbon (POC) content by inhibiting the expression of Endo-1,4-beta-xylanase and Xylan 1,4-beta-xylosidase genes. The accumulation of soil light fraction organic carbon (LFOC) was jointly regulated by multiple microorganisms and enzyme functional genes. *Acaulium*, *Neohelicomyces* and *Ramophialophora* promoted LFOC accumulation by upregulating the expression of genes such as Beta-glucosidase, Endo-1,4-beta-xylanase, Xylan 1,4-beta-xylosidase, peroxidase, Malate dehydrogenase and Pyruvate decarboxylase. Conversely, *Naganishia* and *Tremellomycetes* reduced the LFOC content by inhibiting the expression of some of these genes.

The enrichment patterns of core microorganisms exhibited significant differences across various treatments (Figure S17). The Actinomycetota, *Acaulium*, *Ramophialophora* and *Neohelicomyces* were mostly enriched when using the moldboard tillage (MT) treatment compared to the no-tillage (NT) treatment. Of them, the Actinomycetota and *Acaulium* had a significant enrichment under the specific treatment of full straw return with the use of moldboard tillage (TSMT), whereas the *Neohelicomyces* and the *Ramophialophora* were most abundant under half straw return with moldboard tillage (HSMT). Conversely, no-tillage (NT treatment) significantly enriched *Echria*, *Naganishia*, *Tausonia* and *Tremellomycetes*, with the abundances of these taxa being highest under the full straw return with no-tillage (TSNT).

Soil enzyme activity is one of the main indicators that show the intensity of the processes of soil nutrient cycling. No-tillage treatments, particularly when combined with straw return, significantly increased the activities of soil urease and hydroxylamine reductase (*p* < 0.05). In the process of organic carbon decomposition, moldboard tillage (MT) significantly enhanced the activities of β-glucosidase (BG), β-xylosidase (BX), peroxidase (POD) and polyphenol oxidase (PPO; Figure 4C).

### 3.5. Driving Mechanism of Soil Carbon, Nitrogen and Phosphorus Cycle Based on PLS-PM Model

**Figure 5:**
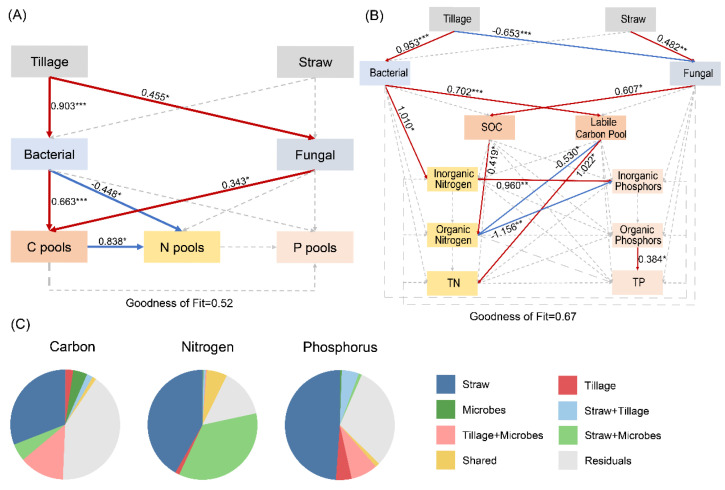
Results of the partial least squares path modeling (PLS-PM) analysis showing how tillage practices and straw return rates drive changes in microbial community structure, thereby influencing soil carbon, nitrogen and phosphorus cycling (**A**,**B**). Variance partitioning analysis (VPA) quantifying the contributions of tillage practices, straw return amount and microbial factors to variations in soil carbon, nitrogen and phosphorus components (**C**). Solid red lines represent significant positive correlations, solid blue lines represent significant negative correlations, and gray dashed lines represent non-significant correlations. Significanct path coefficients are marked with asterisks: * *p* < 0.05, ** *p* < 0.01, *** *p* < 0.001; Bacteria: bacterial Shannon index, PC1 and PC2; Fungi: fungi Shannon index, PC1 and PC2; C pools: SOC, DOC, EOC, POC, HFOC, LFOC, MBC; N pools: TN, NN, AN, AHN, HTN, AAN, ASN, AHAN, UN; P pools: TP, AP, MBP, Resin-P, NaHCO_3_-Pi, NaOH-Pi, D.HCl-Pi, C.HCl-Pi, NaHCO_3_-Po, NaOH-Po, C.HCl-Po, Residual-P; Labile Carbon Pool: POC, DOC, EOC, LFOC, MBC; Inorganic Nitrogen: AN, NN; Organic Nitrogen: AAN, ASN, AHAN, UN; Inorganic Phosphors: AP, TPi; Organic Phosphors: MBP, TPo.

A PLS-PM analysis was employed to elucidate the driving mechanisms through which tillage practices and straw return amounts regulate microbial community structures and subsequently influence soil carbon, nitrogen and phosphorus components. In the model construction, tillage practices were treated as a binary categorical variable (no-tillage = 0 and moldboard tillage = 1), while the straw return amount was set as a three-level continuous variable (no return = 0, half return = 0.5 and full return = 1). The results indicated that moldboard tillage had a direct positive effect on both bacterial and fungal communities (*p* < 0.05). Changes in microbial communities further promoted the accumulation of soil organic carbon components, while both organic carbon and bacterial communities exhibited significant negative effects on soil nitrogen components (*p* < 0.05; Figure 5A). Based on these model outcomes, organic carbon was identified as the key factor driving changes in nitrogen and phosphorus components. Therefore, soil carbon, nitrogen and phosphorus components were further grouped to better investigate the effects of microbially driven organic carbon changes on soil organic, inorganic and total nutrient contents. The results revealed that moldboard tillage significantly promoted the accumulation of soil inorganic nitrogen and labile organic carbon by positively regulating bacterial communities. The growth in the labile organic carbon content had an inhibitory impact on the growth in the organic nitrogen pool but enhanced the total nitrogen content. On the contrary, straw return paired with no-tillage positively controlled fungal communities, which facilitated soil accumulation of total organic carbon (SOC), thus enhancing the ability of soil to store organic nitrogen. There were also notable relationships between the contents of soil nitrogen and phosphorus: inorganic nitrogen helped release inorganic phosphorus whereas organic nitrogen hindered the accretion of inorganic phosphorus (Figure 5B). To obtain a quantification to the relative contributions of different driving factors, a variance partitioning analysis (VPA) was carried out. These findings showed the straw return amount as an independent factor explained a significantly higher proportion of the variation in soil carbon, nitrogen and phosphorus elements as opposed to other factors (Figure 5C).

## 4. Discussion

### 4.1. No-Tillage with Straw Returning Promotes Soil Carbon and Nitrogen Sequestration but Reduces Phosphorus Availability

The carbons, nitrogen and phosphorus components of soil in response to tillage methods and rate of straw return had response characteristics that differed significantly. The straw-returned no-tillage treatments (HSNT, TSNT) had substantial effect on the soil organic carbon (SOC) and microbial biomass carbon (MBC) contents. This is correlated with results of the global-scale research, where the authors claim that the use of straw return was the greatest conservation tillage practice in improving soil carbon sequestration compared to no-tillage and reduced tillage [44]. In no-tillage situations, straw mulching leads to decreasing soil disturbances, as well as lowering the pace of organic carbon mineralization [45]. At the same time, straw input directly offers an exogenous source of carbon, which facilitates the development of the biomass of microbes [46]. Nevertheless, no-tillage treatments had lower content of dissolved organic carbon (DOC), particulate organic carbon (POC) and the light fraction organic carbon (LFOC) as compared to the treatments having moldboard tillage (MT). It can be associated with the increased physical decay of the straw and aggregate turnover caused by tillage [47]. An increase in straw return rate had a positive overall effect on the SOC, MBC content but a negative effect on the POC and easily oxidizable organic carbon (EOC) content. According to this phenomenon, when the rate of straw input is high, it is likely to be converted to stable carbon pools via microbial assimilation as opposed to active carbon fractions [48]. The nitrogen elements analysis showed that no-tilling with the use of straw returns enhanced microbial nitrogen immobilization by means of decreased soil disturbances and the continued supply of carbon sources, which is favorable to the growth of organic nitrogen [15]. In contrast, moldboard tillage contributed to a greater availability of nitrogen through greater release of mineralization processes in soils [49]. The result of this study show that tillage significantly increased the content of soil available phosphorus (AP). This occurred because plowing tillage can promote the transformation of macroaggregates (>0.25 mm) to microaggregates (<0.25 mm). This change in aggregate structure may expose originally physically protected phosphorus components, which are more susceptible to microorganisms and enzymes, and promote the mineralization of organic phosphorus [50]. Straw returning further affects the soil phosphorus pool by directly introducing an organic phosphorus source and stimulating microbial activity, which can affect the content of total phosphorus (TP), organic total phosphorus (TPo) and inorganic total phosphorus (TPi) by regulating microbial activity and phosphatase release [51]. In this study, fungal module 2 was found to be the main microbial cluster that regulates the change in soil phosphorus content, with Penicillium and Aspergillus being significantly enriched in this module under TSMT and HSMT treatments. These two types of microorganisms are recognized as phosphate-solubilizing functional bacteria in soil [52], and their abilities to dissolve insoluble inorganic phosphorus by secreting organic acids and release phosphatase to mineralize organic phosphorus have been confirmed by a large number of studies [53,54,55]. Soil carbon and nitrogen sequestration significantly affects maize growth and farmland economic benefits. No-tillage combined with straw return promotes soil carbon and nitrogen retention, markedly increases the carbon and nitrogen indices in the 0–40 cm soil layer, and thereby facilitates maize growth, development and yield formation [56]. Compared with conventional tillage or straw-free treatments, this practice better regulates the photosynthetic physiological traits of maize and achieves a higher grain yield [57]. Appropriate straw and tillage management strategies not only increase yield but also reduce the carbon footprint by enhancing soil organic carbon (SOC) sequestration, thereby improving the economic and ecological sustainability of agricultural systems [58]. Furthermore, reducing nitrogen fertilizer input by 15–20% under straw return conditions can still maintain high yield and improve nitrogen use efficiency (NUE) while lowering production costs [59]. The analysis of the PERMANOVA measured the extent of the contribution of various factors showing that the straw return rate had a greater influence on the disparity in the rhizosphere nutrient parts than the means of cultivating technique. Research has established a direct effect of straw return on the content of soil carbon, nitrogen and phosphorus through improvement of aggregate-related carbon and microbial biomass carbon [60,61].

### 4.2. Soil Microbial Community Succession Under Conservation Tillage Was Mainly Driven by Carbon and Nitrogen Rather than Phosphorus

Although no-tillage promoted the accumulation of surface carbon, soil compaction inhibited oxygen exchange, resulting in the enrichment of facultative or obligate anaerobic bacteria, the inhibition of aerobic bacteria and a decrease in community richness. In previous studies, no-tillage reduced the uniformity of the microbial community by improving soil stability, enriching specific organic matter decomposition bacteria and reducing low-abundance species [51,62,63]. Significantly correlating with the nutrient content of the soil available, the prevailing phyla in the bacteria included Pseudomonadota, Actinomycetota and Acidobacteriota, which play important functional roles in the cycling of carbon and nitrogen processes in soil [64,65]. In this study, Ascomycota and Basidiomycota were the major fungal communities; TSNT treatment reduced the abundance of Ascomycota while increasing the proportion of Basidiomycota. This could be because the relatively low rate of straw decomposition in no-tillage conditions favors the growth of Basidiomycota, which are also good at degrading lignin and cellulose. On the other hand, Ascomycota, which are more likely tolerant to readily degradable carbon sources, may face competitive limitations under high-straw-return conditions [66,67]. As a result, the TSNT treatment facilitated the development of microbial transformation pathways that were dominated by recalcitrant organic matter, hence maximizing the amount of straw decomposition. The variation partitioning analysis based on a redundancy analysis of bacterial and fungal communities indicated that the succession of the soil microbial community structure was primarily driven by carbon and nitrogen contents, with a relatively minor influence from the phosphorus content. This finding is consistent with several other studies emphasizing that organic carbon and total nitrogen are key variables controlling the microbial community. In other words, the carbon and nitrogen cycles in agricultural soils are highly coupled, jointly serving as the primary substrates and energy sources for microbial metabolism. In contrast, soil phosphorus is mostly present in mineral-bound forms with low bioavailability, exerting only weak direct constraints on community structure [68,69]. Further, the strategy for acquisition of phosphorus by microorganisms is different from that for acquisition of carbon and nitrogen, which mainly depend on the local acidification or enzymatic hydrolysis of specific functional groups rather than the general participation of communities [70,71]. In a straw returning system, the carbon input and the C/N ratio are high, the microorganisms are preferentially limited by nitrogen, and phosphorus is usually not the first limiting factor [72]. These driving relationships have been further proven through random forest model analysis and Mantel tests.

### 4.3. Key Microorganisms Drive Soil Carbon, Nitrogen and Phosphorus Nutrient Cycling by Regulating Enzyme-Related Genes

Microbial networks are very complex and mainly controlled by tillage practices. For instance, it has been discovered that moldboard tillage significantly increases the complexity of bacterial networks. The soil microenvironment changes with the physical disturbance of tillage, which facilitates the dispersal and interaction of bacterial communities by disrupting aggregate structures, increasing interspecies interaction between bacteria, and forming more complex network connections [73]. Conversely, fungal networks have a greater average path length and betweenness centralization under no-tillage conditions, which means that the most significant fungal node microorganisms are more central to the network connectivity under no-tillage conditions [74]. This observation is supported by studies on the expression of functional genes that are under the control of the fungi and which have effects on nutrient cycling. Correlational studies indicate that development of network complexity mainly depends on the bioavailability of nutrients and not on their concentration [75,76].

Microorganisms have specific functional module division, and modular structures promote soil nutrient transformations as a result of synergetic relationships between microorganisms [77]. The ecological functions and roles of bacterial and fungal communities in soil carbon cycling, nitrogen and phosphorus were identified based on a modularity analysis and showed in the present research. Bacterial module 2 is a synergistic unit that facilitates the transformation of nitrogen in soils and the provision of carbon and has the major metabolic functions to be applied in the transformation of ammonia oxidation, biological nitrogen fixation and organic carbon mineralization. Some taxa, including *Nitrososphaeraceae*, can convert ammonium nitrogen into nitrate nitrogen, providing available nitrogen sources for plants [78], and *Xanthobacteraceae* is able to add to the soil nitrogen pool by fixing nitrogen [15]. At the same time, these energy-intensive reactions have energy and carbon skeletons supplied via the organic carbon mineralization process mediated by *Gaiellales* [79]. All these mechanisms are indicative of the ecological plan of microbial assemblage in accomplishing coupled carbon–nitrogen cycling as metabolic interactions. *Sphingomonas* and *Vicinamibacterales* are several taxa that have accumulated in bacterial module 4 and have a close relationship with the carbon cycle. These taxa can also be important in the breakdown of complex organic matter, use of labile carbon sources and soil stabilization of carbon [80,81]. The functional differentiation of fungal modules shows a merged plan that focuses on the carbon cycle and synergistically converts various nutrients. Microbial taxa that predominate the decomposition process and transformation of soil organic carbon are enriched significantly by each fungal module. Moreover, these modules include taxa with multifunctional metabolic capabilities, including *Mortierella* as well as *Pseudogymnoascus*, which are collectively engaged in the transformation and regulation of nutritional elements of nitrogen and phosphorus [82,83]. The most important microbial taxa as derived by the random forest model further explains the synergies of the microbial communities in the various modules to promote the dynamic changes in the soil nutrient cycles. The potential of these microbial taxa is that they can be central participants in complex soil environments influencing the effective transformation and storage of soil carbon, nitrogen and phosphorus [84].

Microorganisms do not simply react to changes in the environment by modifying the community structure in terms of abundance and community composition; they actively shape nutrient cycling pathways by mechanisms that regulate the expression of their own functional metabolic genes [85,86,87]. Under specific environmental stressors or resource conditions, different groups of microbes are selective activators or repressors of the expression of certain functional genes because of their physiological and metabolic characteristics themselves or ecological strategies [88,89,90]. The percentage of carbohydrate-active enzyme genes in Actinomycetota is 16% which confers a strong capacity for decomposing complex organic matter [91]. Full straw return when conventionally tilled is a competitive advantage to the actinomycetes because it enriches them significantly by providing sources of complex organic carbon of high C/N ratios. Actinomycetota adapt their nitrogen metabolism by downregulating the expression of urease genes, which leads to a decrease in soil bio-mass nitrogen (MBN). The process explains the way in which microorganisms can control their nitrogen acquisition and storage strategies by adjusting the efficacy of the main enzyme genes that play a role in transforming nitrogen [65]. With a focus on mineralizing and using organic nitrogen of straw rather than relying on urea hydrolysis, they will satisfy their own nitrogen requirements and mitigate the competition with urea nitrogen, ultimately revamping the short-term paths of the soil nitrogen cycling and the distribution of nitrogen pools [92]. The use of enzyme activity assays showed that moldboard tillage suppressed urease activity, which is consistent with the hypothesis, that actinomycetes downregulate urease gene expression to decrease their reliance on urea nitrogen [93]. Following the process intensity, this observation supports the process of alteration of the nitrogen transformation pathways.

The prominent role of fungi in the carbon cycle is closely related to the abundance of carbohydrate-active enzyme (CAZymes) gene families in their genomes [94]. The expression of these genes is not continuously active but is strictly regulated by substrate induction, carbon source quality and interactions among microorganisms [95,96,97]. Under carbon source stimuli, such as straw input, fungal taxa with corresponding degradation potential can rapidly upregulate the expression of hydrolase genes to decompose polysaccharides in straw, thereby acquiring energy and carbon skeletons and promoting the transformation of soil organic carbon [23]. Conversely, under conservation tillage practices such as no-tillage, soil fungal communities adapt to new ecological niches through metabolic regulation [23]. Research has indicated that no-tillage has great effects on the stimulation of fungal activity through preserving the soil physical structure and amplifies the contribution of fungi to the fungal-to-bacterial necromass ratio via food chain cascade effects, thus benefiting the carbon sequestration role of oligotrophic fungi. No-tillage defines the particular physical and biological conditions of the soil, thereby directionally increasing the yeast-like fungal groups signified by the *Echria*, *Naganishia*, *Tausonia* and *Tremellomyces* [98,99,100,101,102]. These taxa have a bias in their usage of basic carbon sources and their functional benefit causes a modification within the general metabolic pathways of the microbial community. In particular, the expression of the genes encoding enzymes that are important in the lignocellulose breakdown and the related pathways of formate metabolism are repressed, which in turn postpones the breakdown of complex organic matter like the plant residues into the labile intermediate products. Active carbon elements of soil including easily oxidable organic carbon (EOC), particulate organic carbon (POC) and light fraction organic carbon (LFOC) exhibit a decrease in production rates and, as a result, their components are reduced. This process suggests that conservation tillage repressed the breakdown of soil organic carbon by its various mechanisms by shaping specific fungal communities, which enhance the overall carbon sequestration. In contrast to the pathway of carbon preservation through suppressed decomposition functions under no-tillage, fungal taxa enriched in moldboard tillage with straw return, such as *Acaulium*, *Neohelicomyces* and *Ramophialophora*, increases the degradation of the complex organic carbon compounds and recalcitrant organic matter by synergistically upregulating the expression of various hydrolases, redox enzymes and key metabolic enzyme genes. This initiates the soil microbial carbon metabolism [103,104] and overall leads to the accretion of LFOC. The measurements of enzyme activity indicated that the activities of key hydrolases and redox enzymes related to the degradation of complex organic matter were strongly suppressed in the no-tillage condition, which validates the retardation of processes of organic carbon degradation. The results above indicate that no-tillage reduces soil active organic carbon components by suppressing microbial decomposition functions, while moldboard tillage enhances active organic carbon pathways by activating microbial catabolism. However, this study did not identify the microbial pathways through which no-tillage with straw return increases soil total organic carbon (SOC), highlighting a gap in elucidating the mechanisms of carbon sequestration under conservation tillage. Furthermore, current interpretations of microbial functions primarily rely on high-throughput sequencing-based functional predictions and correlation analyses, lacking validation via microbial pure culture studies or isotopic labeling experiments to verify specific microbial functions and causal effects. Unfortunately, functional prediction results based on PICRUSt2 are still underrepresented in soil source sequences, and there are differences between the results with fine classification resolution and metagenomic data [105]. The tool indirectly infers gene abundance through phylogenetic relationships rather than directly determining functional genes [106]. There are also a large number of uncultured microorganisms in black soil, and the lack of related genomes in the reference database may affect the prediction accuracy [105]. In this study, the tillage treatment significantly changed the phylogenetic composition of the microbial community. Moreover, there may be systematic differences in the representativeness of the database between the groups enriched by the no-tillage and tillage treatments, thus introducing prediction bias between treatments. Therefore, multi-omics technologies, such as metagenomics, meta transcriptomics, metabolomics and proteomics, need to be further integrated into future research, with stable isotope probing and targeted cultivation validation to systematically elucidate the key functional genes, differential metabolites and regulatory networks involved in elemental cycling under conservation tillage.

### 4.4. Conservation Tillage Regulates Soil Microbial Communities to Drive Organic Carbon Accumulation and Nitrogen–Phosphorus Transformation

The PLS-PM model revealed the association pathways through which different tillage practices and soil microbial community structure relate to carbon, nitrogen and phosphorus cycling. The results indicate that, under moldboard tillage, changes in the bacterial community are closely associated with soil inorganic nitrogen and labile organic carbon accumulation [60]. Labile organic carbon, as a readily decomposable and rapidly cycling component of soil organic carbon (SOC), exhibits high dynamism and sensitivity to environmental disturbances, serves as a primary energy source and substrate for microbial activity, and plays a critical role in soil nutrient cycling and ecosystem functions [107]. In this study, an increase in labile organic carbon content was accompanied by a decrease in organic nitrogen pool accumulation and yet was significantly and positively associated with total nitrogen contents. In contrast, under no-tillage with straw return conditions, the fungal community was positively associated with soil total organic carbon accumulation, and this association corresponded to a greater capacity for soil organic nitrogen retention. A coupled association was observed between soil nitrogen and phosphorus content: inorganic nitrogen was positively correlated with the release of inorganic phosphorus, whereas organic nitrogen was negatively correlated with its accumulation. These findings suggest that moldboard tillage corresponds to an association pattern dominated by bacterial community changes, accompanied by enhanced organic carbon mineralization and inorganic nutrient release, whereas no-tillage with straw return corresponds to an association pattern dominated by fungal community regulation, linked to greater retention of organic carbon and nitrogen components. However, it is also important to note that, in this study, microbial regulation explained only 52% of the variation in the total soil carbon, nitrogen and phosphorus nutrient pools (GFI = 0.52). This implies that changes in these nutrient pools are not driven solely by microorganisms. In fact, previous studies have shown that factors such as changes in soil temperature, moisture, enzyme activity and root exudates induced by tillage practices also play important roles [108,109,110]. The variance partitioning analysis (VPA) highlighted the dominant role of straw return in nutrient cycling and found that it likely influences nutrient dynamics through two pathways: direct physical input and chemical stimulation [111]. In contrast, tillage practices primarily exert indirect effects by altering microbial community structures.

## 5. Conclusions

This study revealed the key pathways of conservation tillage that regulate soil carbon, nitrogen and phosphorus cycles through rhizosphere microbial communities. There were significant differences in microbial community structure and nutrient cycling characteristics between no-tillage and tillage straw returning treatments. The bacterial and fungal network modules showed a clear functional division of labor, with specific microbial modules significantly affecting soil nutrient dynamics by enriching the core groups of nitrogen transformation, carbon degradation and phosphorus activation. Therefore, differentiated farming strategies should be adopted according to local conditions in typical black soil areas: In areas with high erosion risk or poor soil fertility, long-term no-tillage and full straw returning treatments should be promoted to strengthen fungal-mediated carbon and nitrogen sequestration. In areas with good soil fertility, it is advisable to adopt a rotation mode combining no-tillage and tillage straw returning treatments to release available nutrients by intermittent activation of bacterial communities. The directional regulation of key microbial communities by optimizing tillage and straw management is of great significance for the cultivation and sustainable protection of black soil fertility.

## Data Availability

The raw sequences were submitted into the Sequence Read Archive (SRA) database (Accession Number: PRJNA1399928).

## Supplementary Materials

The following supporting information can be downloaded at https://www.mdpi.com/article/10.3390/microorganisms14051092/s1, Figure S1: Variation in soil carbon fractions under different tillage practices; Figure S2: Variation in soil nitrogen fractions under different tillage practices; Figure S3: Variation in soil phosphorus fractions under different tillage practices; Figure S4: (A) Alpha diversity of soil bacteria and fungi under different tillage practices; Figure S5: Composition of Soil Bacterial (A) and Fungal (B) Communities at the Phylum Level under Different Tillage Practices; Figure S6: Relationships between the abundance of dominant soil bacterial (A) and fungal (B) phyla and soil carbon, nitrogen, and phosphorus fractions; Figure S7: Mantel analysis of the relationships between bacterial and fungal communities and soil carbon, nitrogen, and phosphorus fractions; Figure S8: (A) Bacterial and fungal interaction network constructed from all rhizosphere soil samples; (B) Bacterial network under different treatments; (C) Fungal network under different treatments; Figure S9: Topological properties of bacterial co-occurrence networks; Figure S10: Topological properties of fungal co-occurrence networks; Figure S11: Correlation heatmap between topological properties of bacterial and fungal networks and soil carbon, nitrogen, and phosphorus fractions; Figure S12: (A) Modular structure of the bacterial community co-occurrence network. The network was partitioned into four modules (labeled 1 to 4), containing 153, 92, 94, and 1155 microbial taxa at the genus level, respectively; (B) Modular structure of the fungal community co-occurrence network; Figure S13: Top 10 genera ranked by microbial abundance within bacterial and fungal modules; Figure S14: Identification of Key Bacterial Taxa Influencing Nutrient Changes Using a Random Forest Model; Figure S15: Identification of Key Fungal Taxa Influencing Nutrient Changes Using a Random Forest Model; Figure S16: Pathway analysis of how core microorganisms mediate nutrient cycling by regulating the expression of functional enzyme genes; Figure S17: Abundance of Core Soil Microorganisms and Analysis of Variance under Different Tillage Practices; Table S1: Differences in soil nutrient contents and plant phenotypes between different treatments in 2022 and 2023; Table S2: Sequences of the primers for bacteria and fungi; Table S3: Bacterial PICRUSt2 prediction results functional annotation; Table S4: Fungal PICRUSt2 prediction results functional annotation. Text S1: Soil physicochemical examination, Text S2: Soil microbial detection, Text S3: Detection of soil enzyme activity.
