# Supplementary material for: Conservation Tillage-Mediated Rhizosphere Microbial Community Remodeling Drives Soil Organic Carbon Accumulation and Nitrogen and Phosphorus Transformation in Farmland
Source: Microorganisms. 2026 May 12;14(5):1092. doi: 10.3390/microorganisms14051092 (PMC13210006; doi:10.3390/microorganisms14051092)
Supplement: Supplementary file 1 [file microorganisms-14-01092-s001.zip › microorganisms-4285926-supplementary.pdf]

Supplementary Materials

Title: Conservation Tillage-Mediated Rhizosphere Microbial Community Remodeling Drives Soil Organic Carbon Accumulation and Nitrogen and Phosphorus Transformation in Farmland

Names of authors: Haogeng Zhao [1,2], Meijuan Cheng [3], Shuli Wei [2], Gongfu Shi [2], Jing Fang [3], Huimin Shi [2], Qingze Liu [1], Yan Qu [2], Weijing Zhang [3], Fang Luo [4], Yu Wang [4], Zhanyuan Lu [2], Dejian Zhang [1,*] and Xiaoqing Zhao [2,*]

Affiliations: [1] *School of Life Science, Inner Mongolia University, Hohhot 010020, China*

[2] *Inner Mongolia Academy of Agricultural & Animal Husbandry Sciences, Key Laboratory of Black Soil Protection and Utilization/Inner Mongolia Key Laboratory of Degradation Farmland Ecological Restoration and Pollution Control, Hohhot 010031, China*

[3] *College of Agronomy, Inner Mongolia Agricultural University, Hohhot 010018, China*

[4] *Arun Banner Agricultural Technology Promotion Center, Hulun Buir 162750, China*

Corresponding author: Dejian Zhang, Xiaoqing Zhao

E-mail address: zhangdejian00@163.com (D.Z.); zhaoxq204@163.com (X.Z.)

Text S1 Soil physicochemical examination

Soil organic carbon (SOC) content was determined using the dichromate oxidation method. The following methods were employed for the determination of soil organic carbon fractions: easily oxidizable organic carbon (EOC) was determined using the potassium permanganate oxidation method; dissolved organic carbon (DOC) was extracted with potassium sulfate (K_2SO_4) solution and measured accordingly; particulate organic carbon (POC) was quantified by the potassium dichromate volumetric method with external heating; heavy fraction organic carbon (HFOC) was determined via the potassium dichromate heating method, and light fraction organic carbon (LFOC) content was calculated by subtracting HFOC from the total organic carbon content. Total nitrogen (TN) content was analyzed following the Kjeldahl digestion method. For the determination of soil nitrate (NO_3^--N) and ammonium (NH_4^+-N) content, soil samples were extracted with 1 mol L^{-1} KCl solution (1:10 w/v)) ratio, and the extracts were subsequently analyzed using a continuous flow injection analyzer (FLOWSYS, Italy). Soil alkaline hydrolyzable nitrogen (AHN) was determined by the sodium hydroxide hydrolysis-diffusion method. Soil organic nitrogen fractions were analyzed using the Bremner method. Specifically, hydrolyzable total nitrogen (HTN) was measured by acid hydrolysis with 6 mol/L HCl followed by the Kjeldahl method; acid-hydrolyzable ammonium nitrogen (AHAN) was determined by distillation with 3.5% MgO; the sum of acid-hydrolyzable ammonium nitrogen and amino sugar nitrogen (AHAN + ASN) was quantified by distillation with a phosphate-borate buffer (pH = 11.2); amino acid nitrogen (AAN) was analyzed using ninhydrin oxidation followed by distillation with a phosphate-borate buffer. Acid-hydrolyzable unknown nitrogen (UN) and amino sugar nitrogen (ASN) were calculated by difference, using the formulas: UN = HTN - AHAN - AAN - ASN and ASN = (AHAN + ASN) - AHAN.Total phosphorus (TP) content was measured through sulfuric acid–perchloric acid digestion, followed by the ascorbic acid–molybdophosphate blue method. Available phosphorus (AP) was quantified using the sodium bicarbonate ($NaHCO_3$) extraction method. Soil phosphorus fractionation was determined using the improved Hedley phosphorus fractionation method by Tiessen et al. A 0.5 g soil sample was sequentially extracted to determine resin-extractable phosphorus (Resin-P), $NaHCO_3$-extractable phosphorus ($NaHCO_3$-Pi + $NaHCO_3$-Po), NaOH-extractable phosphorus (NaOH-Pi + NaOH-Po), dilute HCl-extractable phosphorus (D.HCl-Pi), and concentrated HCl-extractable phosphorus

(C.HCl-Pi and C.HCl-Po), as well as residual phosphorus (Residual-P). The sum of Resin-P, $NaHCO_3$-Pi, NaOH-Pi, D.HCl-Pi, and C.HCl-Pi represents the total inorganic phosphorus content (TPi), while the sum of $NaHCO_3$-Po, NaOH-Po, and C.HCl-Po represents the total organic phosphorus content (TPo).Soil microbial biomass carbon (MBC), nitrogen (MBN), and phosphorus (MBP) were determined using the chloroform fumigatio–extraction method.

Text S2 Soil microbial detection

Total DNA was extracted from 0.05 g of rhizosphere soil samples using the FastDNA® SPIN Kit for Soil (MP Biomedicals, USA). The bacterial 16S rRNA gene was amplified with the primer set 515F/806R, and the fungal ITS1 region was amplified using primers ITS1F/ITS2R (Table S1). The PCR reaction mixture had a total volume of 20 μL, containing 4 μL of 5× buffer (replaced with 2 μL of 10× buffer for ITS1 amplification), 0.8 μL of each primer, 2 μL of 2.5 mM dNTPs, 10 ng of DNA template, 0.4 μL of FastPfu polymerase (replaced with 0.2 μL of Taq polymerase for ITS1 amplification), 0.2 μL of BSA, and ddH_2O to a final volume of 20 μL. The amplification products were recovered via 2% agarose gel electrophoresis, purified using the AxyPrep DNA Gel Extraction Kit, and quantified with the Quantus™ Fluorometer. Purified amplicons were subjected to paired-end sequencing on the Illumina HiSeq PE2500 platform by Majorbio (Shanghai). Raw sequences were processed using USEARCH for assembly, followed by removal of short sequences (<200 bp) and primer sequences. UPARSE was used to cluster operational taxonomic units (OTUs) at 97% similarity, and UCHIME was applied for chimera removal. Low-quality sequences ($Q < 20$) were filtered using QIIME2, and paired-end reads were merged with FLASH. Final OTUs were generated at 97% similarity. Taxonomic classification of bacterial 16S rRNA and fungal ITS rRNA OTUs was performed using the Greengenes database and the SILVA database, respectively, as reference sequences.

Text S3 Detection of soil enzyme activity

Soil urease (Ure) activity was determined using the indophenol blue colorimetric method. Soil hydroxylamine reductase (HyR) activity was determined using the o-phenanthroline colorimetric method, with one unit of enzyme activity defined as the amount of enzyme that converts 1 μmol of hydroxylamine per day per gram of soil. Glutaminase activity was measured using Nessler's colorimetric method. β-Glucosidase (BG) catalyzes the hydrolysis of p-nitrophenyl-β-D-glucopyranoside to produce p-nitrophenol, which exhibits a faint yellow color and has a characteristic absorption peak at 400 nm. The activity of β-xylosidase (BX) was determined using a microplate-based enzymatic assay with ultraviolet fluorescence detection. Polyphenol oxidase (PPO) activity was determined by the pyrogallol colorimetric method. Polyphenol oxidase (PPO) and peroxidase (POD) activities were determined using the pyrogallol colorimetric method.

Table S1 Differences in soil nutrient contents and plant phenotypes between different treatments in 2022 and 2023.

Year	Indicator	NSMT	HSMT	TSMT	NSNT	HSNT	TSNT
2022	SOC	26.6±1.02bc	23.94±2.52c	27.49±0.67 b	28.6±0.39 b	38.72±0.00a	36.85±2.56a
	TN	2.32±0.03d	2.17±0.08e	2.13±0.07e	2.88±0.04a	2.51±0.04c	2.75±0.05b
	AN	11.15±0.93bc	13.79±1.53 a	11.43±0.93bc	9.81±0.68c	9.94±0.7c	12.64±0.41ab
	TP	0.68±0.19a	0.46±0.13b	0.63±0.1ab	0.2±0.02c	0.57± 0.1ab	0.69±0.04a
	AP	7.01±0.81a	5.02±0.71b	6.85±1.29a	3.63±0.23bc	3.26±1.14c	2.39±0.33c
	PH	209±2.65bc	227.33±8.96a	215±1.00b	191.6±6.99d	198.2±3.52d	201.5±5.21cd
	FW	647.15±94.8b	840.48±52.5a	835.09±95.6a	902.15±31.2a	957.15±1a	917.15±25.9a
2023	SOC	24.16±0.67c	28.15±1.76b	29.04±0.38b	26.9±1.68b	41.45±1.76a	41.23±1.02a
	TN	2.07±0.02c	2.04±0.04c	2.05±0.08c	2.45±0.07a	2.4±0.09a	2.29±0.05b
	AN	7.82±0.69c	10.76±0.85b	15.43±1.22a	7.02±0.97c	8.07±1.85c	7.58±1.26c
	TP	0.52±0.07abc	0.55±0.07 b	0.64±0.1a	0.47±0.12bc	0.51±0.11abc	0.37±0.02c
	AP	4.55±0.56b	5.54±0.49a	4.06±0.23b	2.52±0.29c	2.99±0.46c	1.72±0.31d
	PH	210±3.00d	227±3.61ab	232±2.65a	218.67±1.53c	231.67±3.51a	224.67±2.31b
	FW	485.48±72.51b	548.82±133.82b	792.15±147.73a	759.3±81.29a	832.64±110.15a	759.3±81.29a

Table S2. Sequences of the primers for bacteria and fungi.

Soil microbes	Primer code	Primer sequence
Bacteria	515F	5′-GTGCCAGCMGCCGCGGTAA-3′
	806R	5′-GGACTACHVGGGTWTCTAAT-3′
Fungi	ITS1F	5′-CTTGGTCATTTAGAGGAAGTAA-3′
	ITS2R	5′-GCTGCGTTCTTCATCGATGC-3′

Table S3 Bacterial PICRUSt2 prediction results functional annotation

ID	Functional gene name	Mean abundance					
		NSMT	HSMT	TSMT	NSNT	HSNT	TSNT
3.2.1.4	Cellulase	19891.9	19452.94	19012.26	18930.12	17693.78	19941.52
3.2.1.21	Beta-glucosidase	50978.96	53025.85	50438.18	50077.65	43769.2	59850.46
3.2.1.91	Cellulose 1,4-beta-cellobiosidase	351.02	376.92	324.64	273.6933	243.9467	519.8933
3.2.1.37	Xylan 1,4-beta-xylosidase	2419.717	2819.917	2684.143	2851.74	2084.637	3574.113
3.2.1.15	Polygalacturonase	563.4433	537.7767	550.89	464.3333	374	713.3867
3.1.1.11	Pectinesterase	1360.16	2140.58	1253.207	1929.713	1368.23	1602.91
3.2.1.1	Alpha-amylase	17566.78	17605.92	16886.57	17495.38	16344.13	18700.15
3.2.1.3	Glucan 1,4-alpha-glucosidase	1462.417	1411.183	1354.163	1174.457	1252.627	1552.097
1.1.1.90	Aryl-alcohol dehydrogenase	2692.94	2110.903	1890.727	1650.597	1797.417	4584.24
1.13.11.1	Catechol 1,2-dioxygenase	3261.987	3233.923	2718.33	2755.083	2500.603	4729.943
1.13.11.3	Protocatechuate 3,4-dioxygenase	13811.54	14224.38	13098.03	11709.47	10390.79	16604.61
1.14.18.3	Methane monooxygenase (particulate)	2413.167	2347.11	1909.833	2938.167	4332.667	2416.833
1.1.2.7	Methanol dehydrogenase (cytochrome c)	383.2667	449.22	402	433	312.6667	404.6667
4.1.1.39	Ribulose-bisphosphate carboxylase	5151.7	5709.603	5521.533	5052.99	4725.57	6127.34
4.1.3.1	Isocitrate lyase	7564.387	8671.69	7937.963	8253.203	6557.697	10798.62
4.1.1.31	Phosphoenolpyruvate carboxylase	17204.4	17594.94	17698.73	18232.27	16363.89	18411.48
1.1.1.27	L-lactate dehydrogenase	1888.787	1952.107	1673.57	1946.447	1941.383	2116.56
2.7.2.1	Acetate kinase	20790.44	19050.19	20273.2	19886.23	20431.25	19650.74
4.2.1.2	Fumarate hydratase	44809.34	45470.11	45705.14	44028.04	41745.5	44917.16
1.18.6.1	Nitrogenase	2383.583	2648.133	2514.9	2484.13	2205.08	3521.093
1.14.99.39	Ammonia monooxygenase	2413.167	2347.11	1909.833	2938.167	4332.667	2416.833
1.7.2.6	Hydroxylamine dehydrogenase	28	30.11	34.66667	50.66667	103.6667	59.66667
1.7.2.1	Nitrite reductase (NO-forming)	7896.543	8399.183	8136.073	9588.757	11429.73	8768.823
1.7.2.5	Nitric-oxide reductase (cytochrome c)	1647.63	1890.19	2287.567	2184.233	1352.077	2357.91
1.7.2.4	Nitrous-oxide reductase	2240.877	2815.057	2836.657	3213.333	2471.78	3088.557
1.7.99.4	Nitrate reductase	29814.97	28364.96	28562.1	27871.77	28704.49	29866.95
3.5.1.5	Urease	28508.47	29961.15	26111.63	29302.28	30384.54	35689.77
3.5.1.2	Glutaminase	4535.797	4530.713	3964.7	4147.977	3611.11	6386.467
3.4.11.1	Leucyl aminopeptidase	36429.91	37649.75	37691.12	36224.83	35230.84	39329.77
1.4.1.13	Glutamate synthase (NADPH)	50442.09	52076.45	52289.54	49830.03	46405.02	53555.82
1.4.1.14	Glutamate synthase (NADH)	50442.09	52076.45	52289.54	49830.03	46405.02	53555.82
6.3.1.2	Glutamate--ammonia ligase	65580.35	66269.59	64729.78	62725.06	62382.01	69699.91
3.1.3.1	Alkaline phosphatase	24244.35	25675.98	24834.8	24841.26	22293.05	25610.98
3.1.3.2	Acid phosphatase	4298.963	4707.88	4659.577	4198.017	3499.937	5716.63

3.1.3.8	3-phytase	2111.537	2932.903	2631.73	2522.82	1865.867	2546.547
3.1.3.26	4-phytase	1901.58	1781.027	1611.167	1577.16	1675.55	2017.903
3.1.4.3	Phospholipase C	12159.33	12780.22	11542.26	11186.22	10520.35	15223.97
3.1.4.4	Phospholipase D	64	73.83333	67	91.83333	54.66667	132.8333
2.7.4.1	Polyphosphate kinase	19716.04	20677.63	20834.74	21240.2	18533.8	22695.41
3.6.1.11	Exopolyphosphatase	35092.22	36535.65	36159.1	34370.79	31804.2	38544.59
3.6.3.27	Phosphate-transporting ATPase	36188.35	35622.83	35840.4	34995.95	35224.39	34798.78

Table S4 Fungal PICRUSt2 prediction results functional annotation

ID	Functional gene name	Mean abundance					
		NSMT	HSMT	TSMT	NSNT	HSNT	TSNT
3.2.1.4	Cellulase	43362.67	49254.33	60403.67	49379.17	43423.5	48491.67
3.2.1.21	Beta-glucosidase	857477.3	863205.9	858273.7	820731.9	803921.5	848839.7
3.2.1.8	Endo-1,4-beta-xylanase	9341	10503.33	11307.67	8184.833	3961.167	3329.333
3.2.1.37	Xylan 1,4-beta-xylosidase	9514.667	11356	13804.67	11692.5	4729.5	3761
1.10.3.2	Laccase	317580.5	318421.4	324506.9	338091.6	326075.7	344380.8
1.11.1.7	Peroxidase	44641.38	42658.09	36320.39	36572.73	31535.17	31166.62
1.13.11.1	Catechol 1,2-dioxygenase	12518	12316.67	11727	11771.17	7079.5	7528
1.1.1.49	Glucose-6-phosphate dehydrogenase (NADP(+))	71941.86	72536.05	71610.53	70831.12	72165.17	70887.81
1.1.1.44	Phosphogluconate dehydrogenase(NADP(+)-dependent, decarboxylating)	81261.71	80213.09	79841.72	84700.9	79222	82664.29
1.1.1.37	Malate dehydrogenase	164183.8	163981.5	160223.1	154332.5	155848	156008.9
4.1.1.1	Pyruvate decarboxylase	121711	120943.4	114890.7	111876.7	107043.2	106599.6
1.2.1.2	Formate dehydrogenase	67603.05	68320.76	68767.72	68708.4	68182.83	69604.95
6.3.1.2	Glutamate--ammonia ligase	165832.6	167282.5	177461.6	168970.5	170044.7	177308.1
3.4.11.1	Leucyl aminopeptidase	22139.67	23594.33	30573.33	27093.33	31953.67	38919
3.5.1.5	Urease	71052.52	70545.05	71504.86	70807.12	70375.17	70446.81
3.2.1.14	Chitinase	669691.1	673160.3	690017.2	647781.9	671472.2	719333.5
1.4.1.2	Glutamate dehydrogenase	69922.52	69962.38	70009.53	70012.45	70034.17	70025.48
2.6.1.1	Aspartate transaminase	230530	232032.9	230681.6	226830.4	226602.3	222207.7
3.1.3.1	Alkaline phosphatase	118756.7	113471.8	108379.4	101419.4	90965.17	78062.95
3.1.3.2	Acid phosphatase	467675.4	468555.8	480372.1	464987.7	455965.8	485740.6
3.1.3.8	3-phytase	14084	15670.67	16240.33	12514.67	7389	6773.667
3.6.1.11	Exopolyphosphatase	71915.19	72919.05	72931.19	76197.28	72694.67	73355.14
3.6.1.3	Adenosinetriphosphatase	3539642	3524727	3532622	3532216	3612042	3608517
3.1.4.1	Phosphodiesterase I	119301.9	119139.7	122892.5	122477.6	126144	126287.5
3.1.4.3	Phospholipase C	5497.667	6347	8753	2777.333	1460	1636.667
3.1.4.4	Phospholipase D	209648.9	208768.8	207576.9	203930.2	206237.7	210049.8

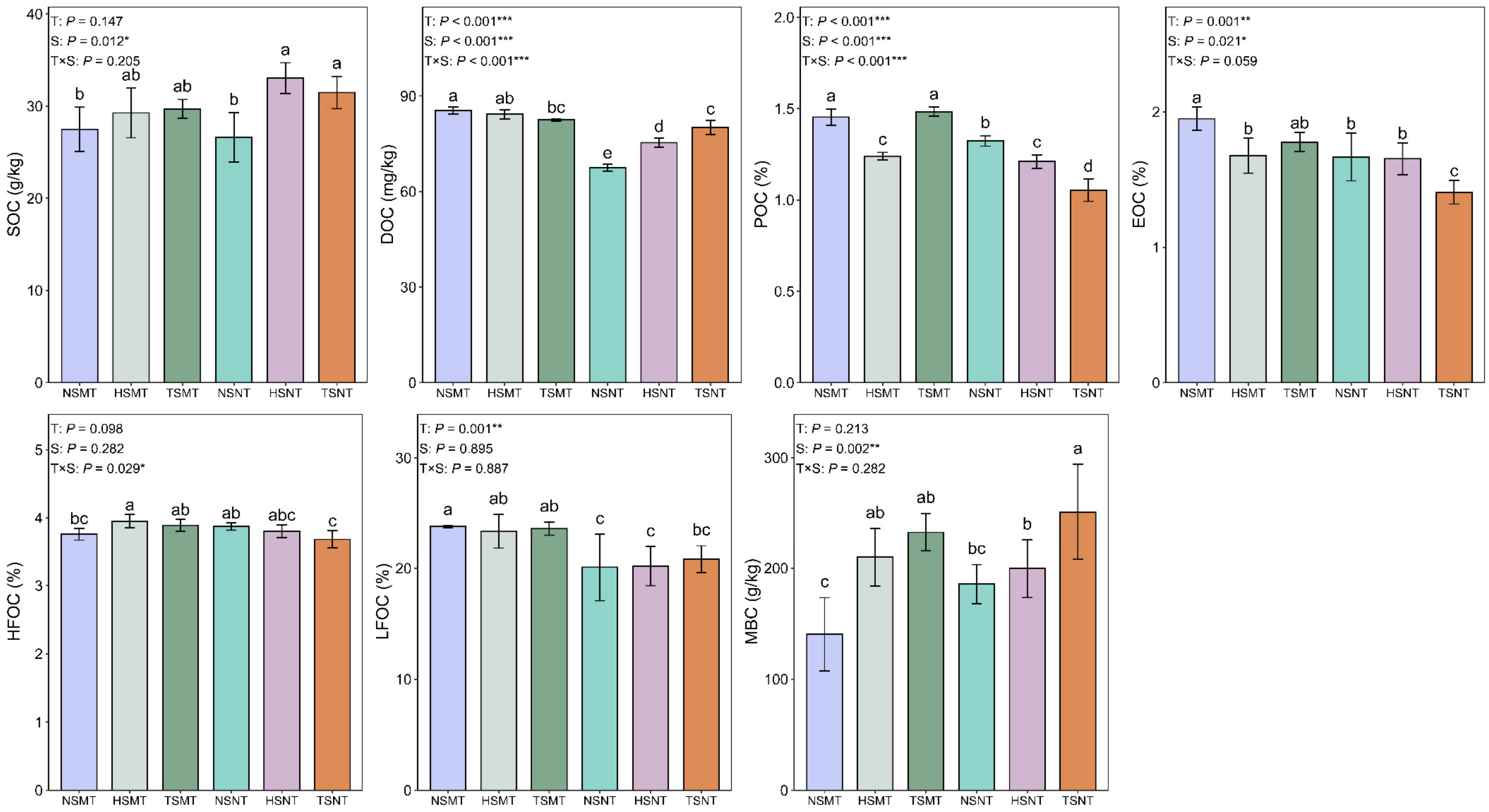

Figure S1. Variation in soil carbon fractions under different tillage practices. T: Tillage, S: Straw; Different lowercase letters above boxplots indicate significant differences among treatments ($p < 0.05$);

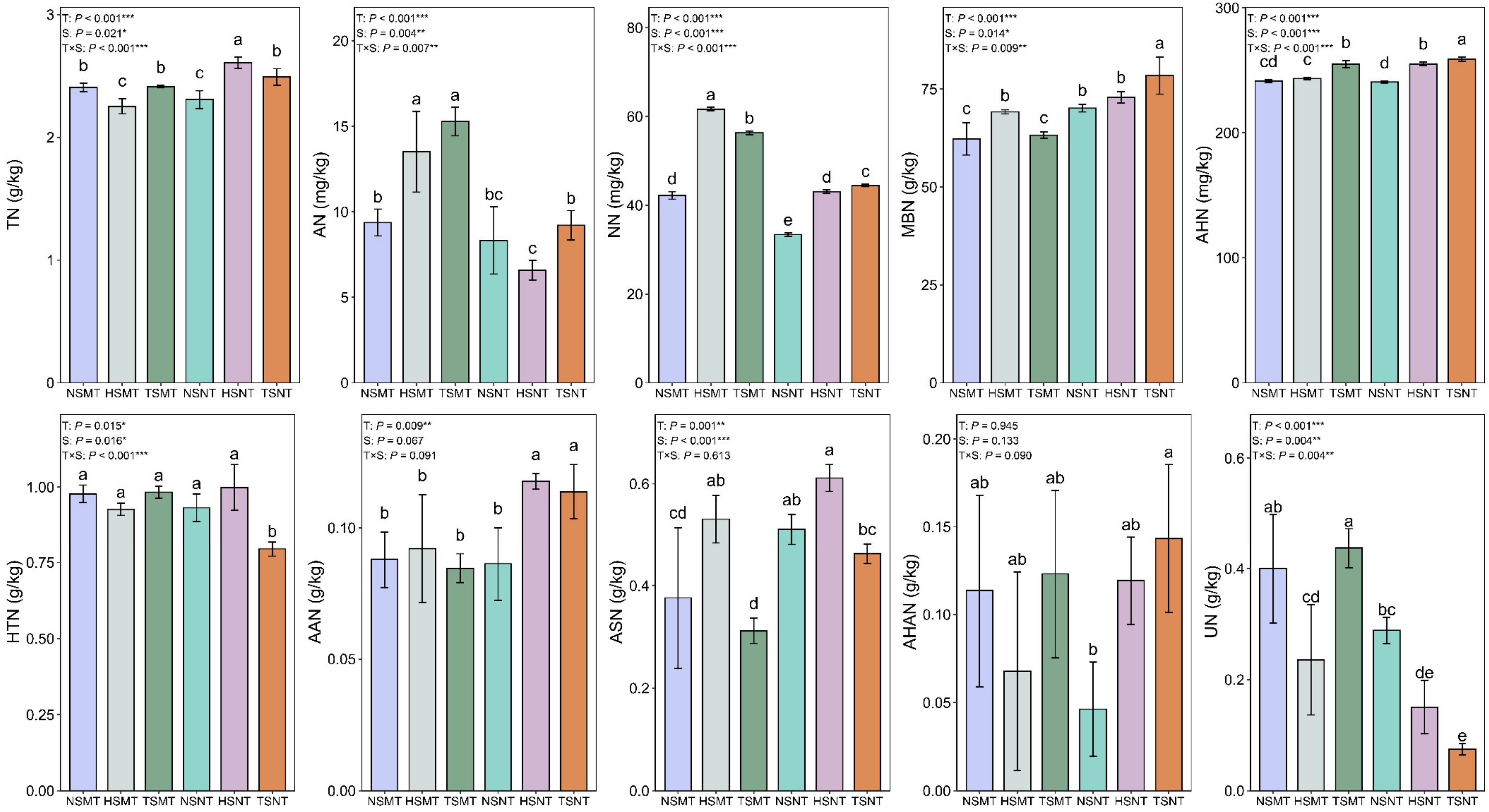

Figure S2. Variation in soil nitrogen fractions under different tillage practices. T: Tillage, S: Straw; Different lowercase letters above boxplots indicate significant differences among treatments ($p < 0.05$);

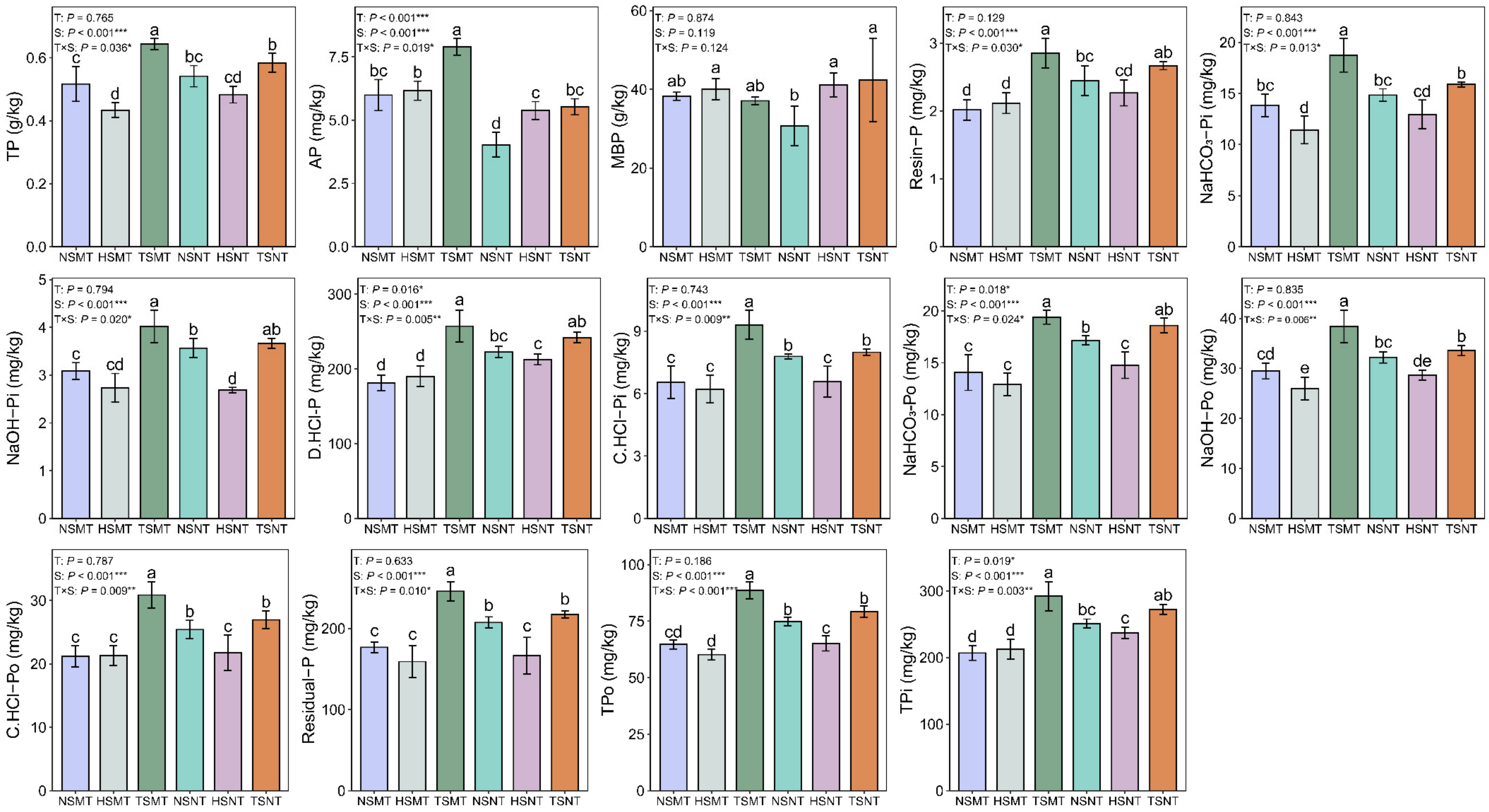

Figure S3. Variation in soil phosphorus fractions under different tillage practices. T: Tillage, S: Straw; Different lowercase letters above boxplots indicate significant differences among treatments ($p < 0.05$);

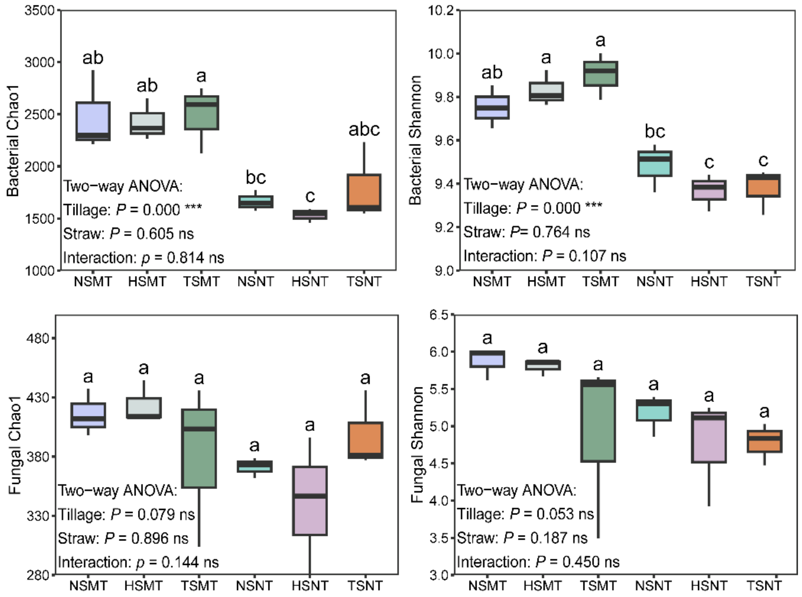

Treatment	Bacterial Chao1	Bacterial Shannon	Fungal Chao1	Fungal Shannon
NSMT	2477.58±386.74	9.75±0.10	415.73±19.86	5.87±0.22
HSMT	2427.872±200.72	9.83±0.08	423.79±17.91	5.80±0.11
TSMT	2487.763±325.08	9.90±0.11	381.08±68.57	4.90±1.22
NSNT	1664.192±99.94	9.48±0.11	371.17±8.44	5.18±0.29
HSNT	1531.666±68.77	9.37±0.09	341.26±57.77	4.76±0.73
TSNT	1795.77±378.06	9.38±0.10	397.93±32.88	4.78±0.28

Figure S4. (A) Alpha diversity of soil bacteria and fungi under different tillage practices. Different lowercase letters above boxplots indicate significant differences among treatments ($P < 0.05$).

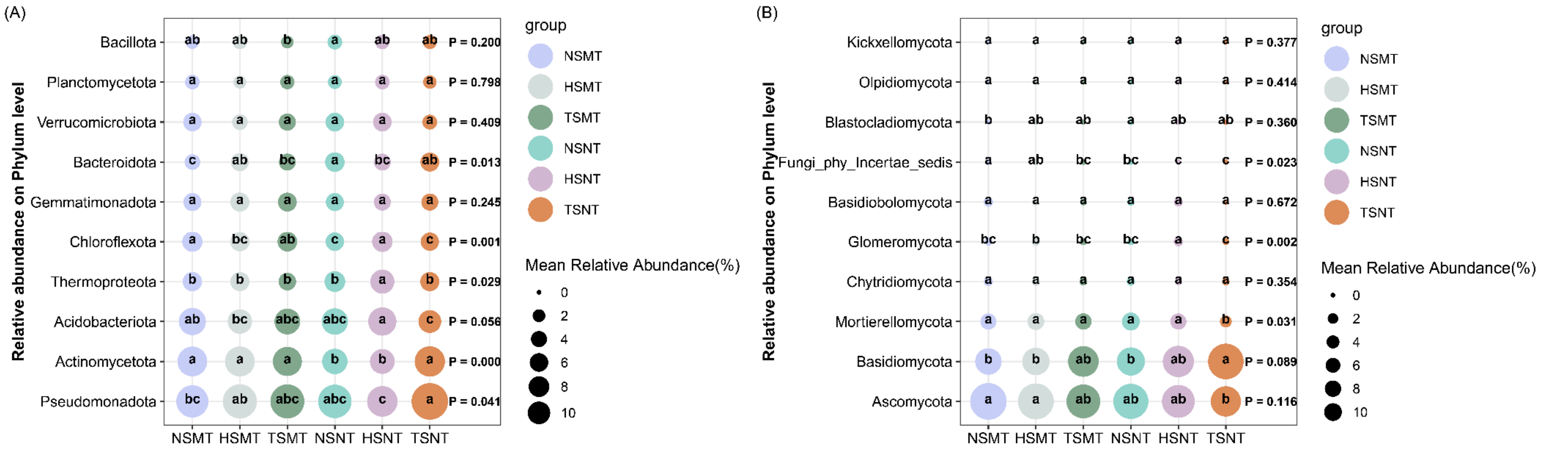

Figure S5. Composition of Soil Bacterial (A) and Fungal (B) Communities at the Phylum Level under Different Tillage Practices. Different lowercase letters above boxplots indicate significant differences among treatments ($p < 0.05$);

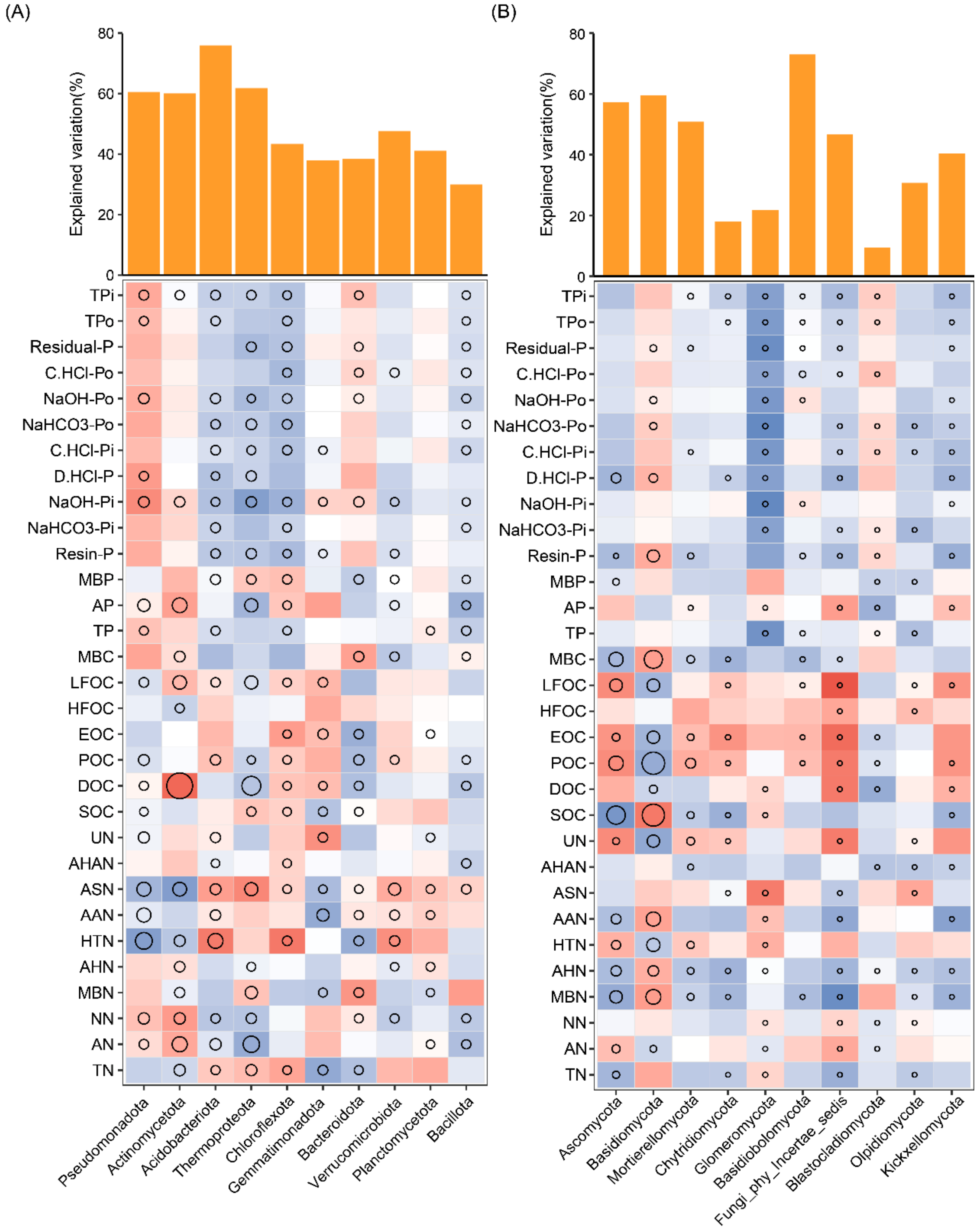

Figure S6. Relationships between the abundance of dominant soil bacterial (A) and fungal (B) phyla and soil carbon, nitrogen, and phosphorus fractions.

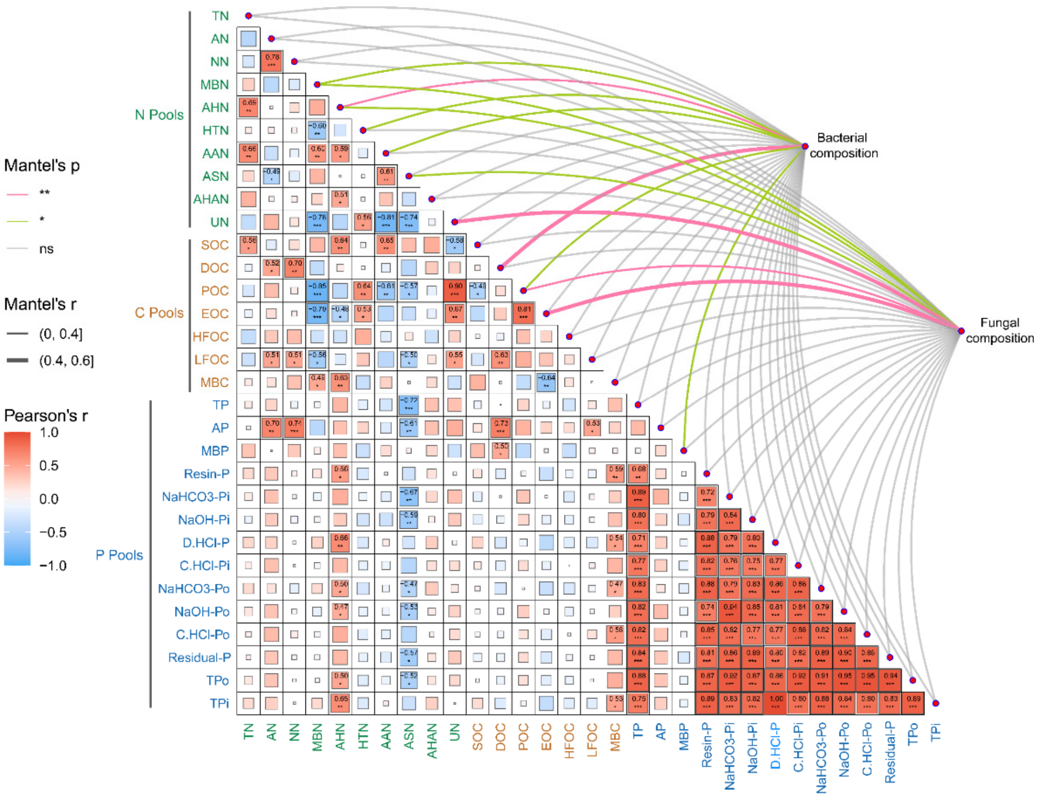

Figure S7. Mantel analysis of the relationships between bacterial and fungal communities and soil carbon, nitrogen, and phosphorus fractions.

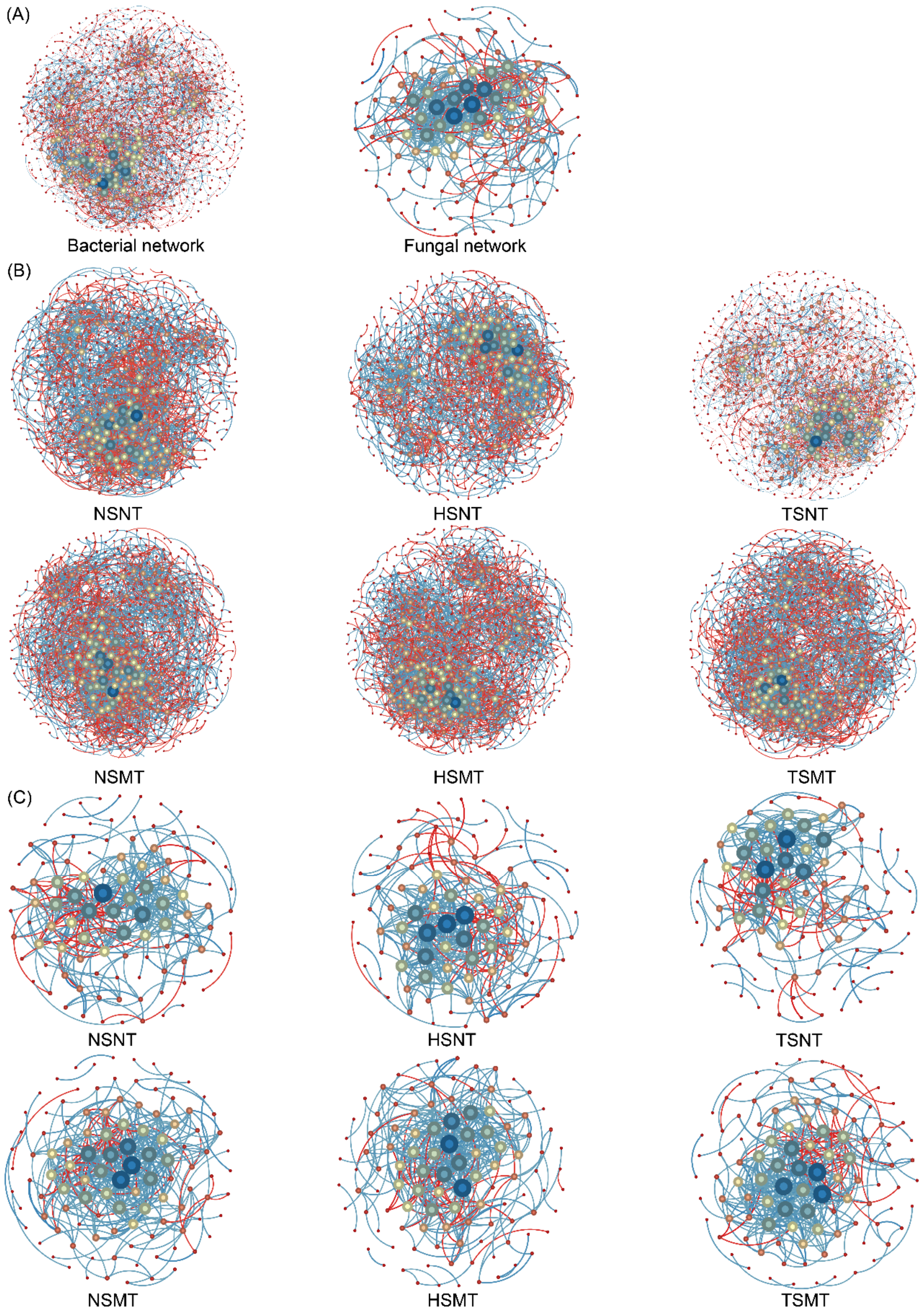

Figure S8. (A) Bacterial and fungal interaction network constructed from all rhizosphere soil samples; (B) Bacterial network under different treatments; (C) Fungal network under different treatments.

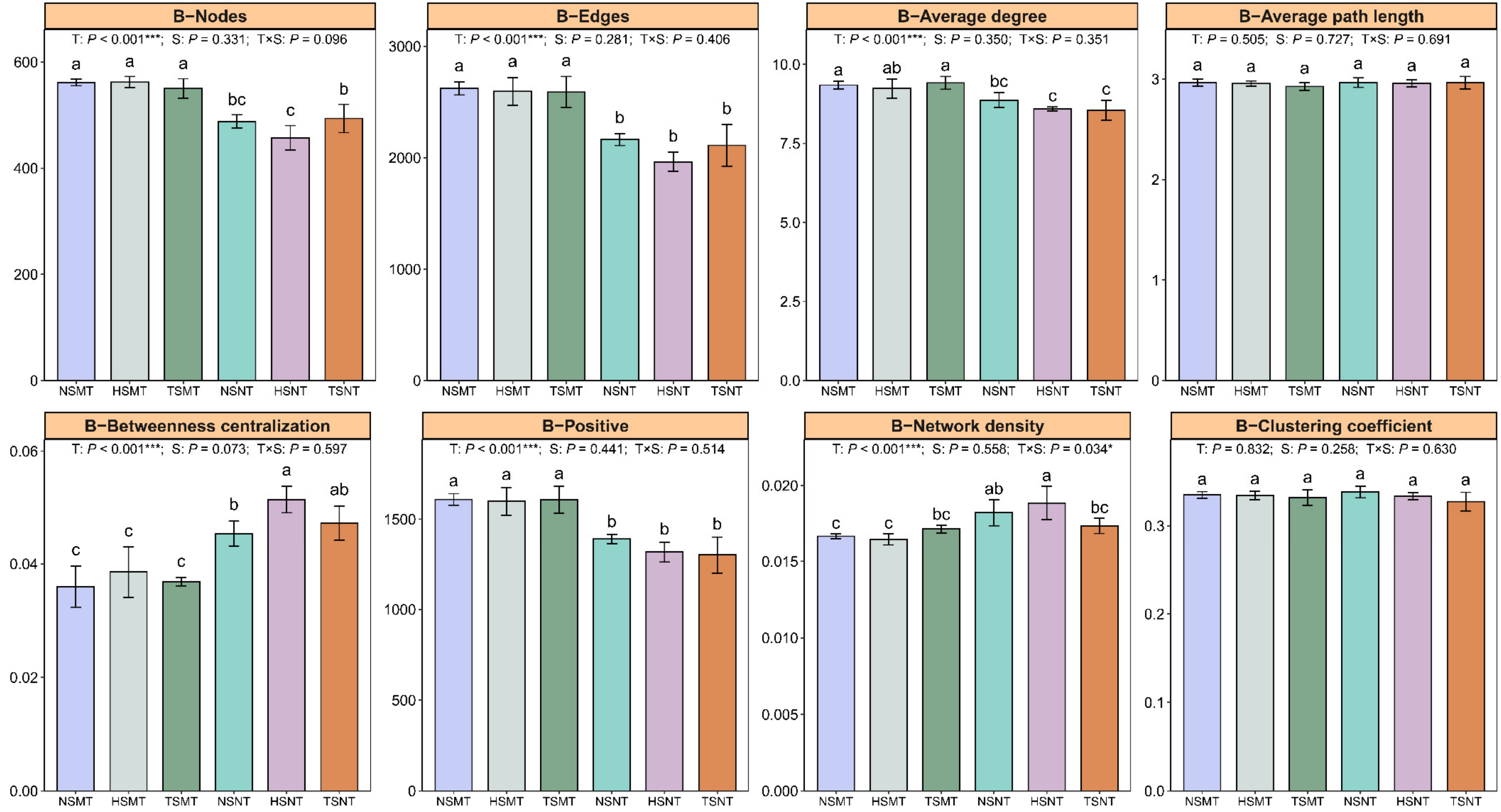

Figure S9. Topological properties of bacterial co-occurrence networks. Different lowercase letters above boxplots indicate significant differences among treatments ($p < 0.05$).

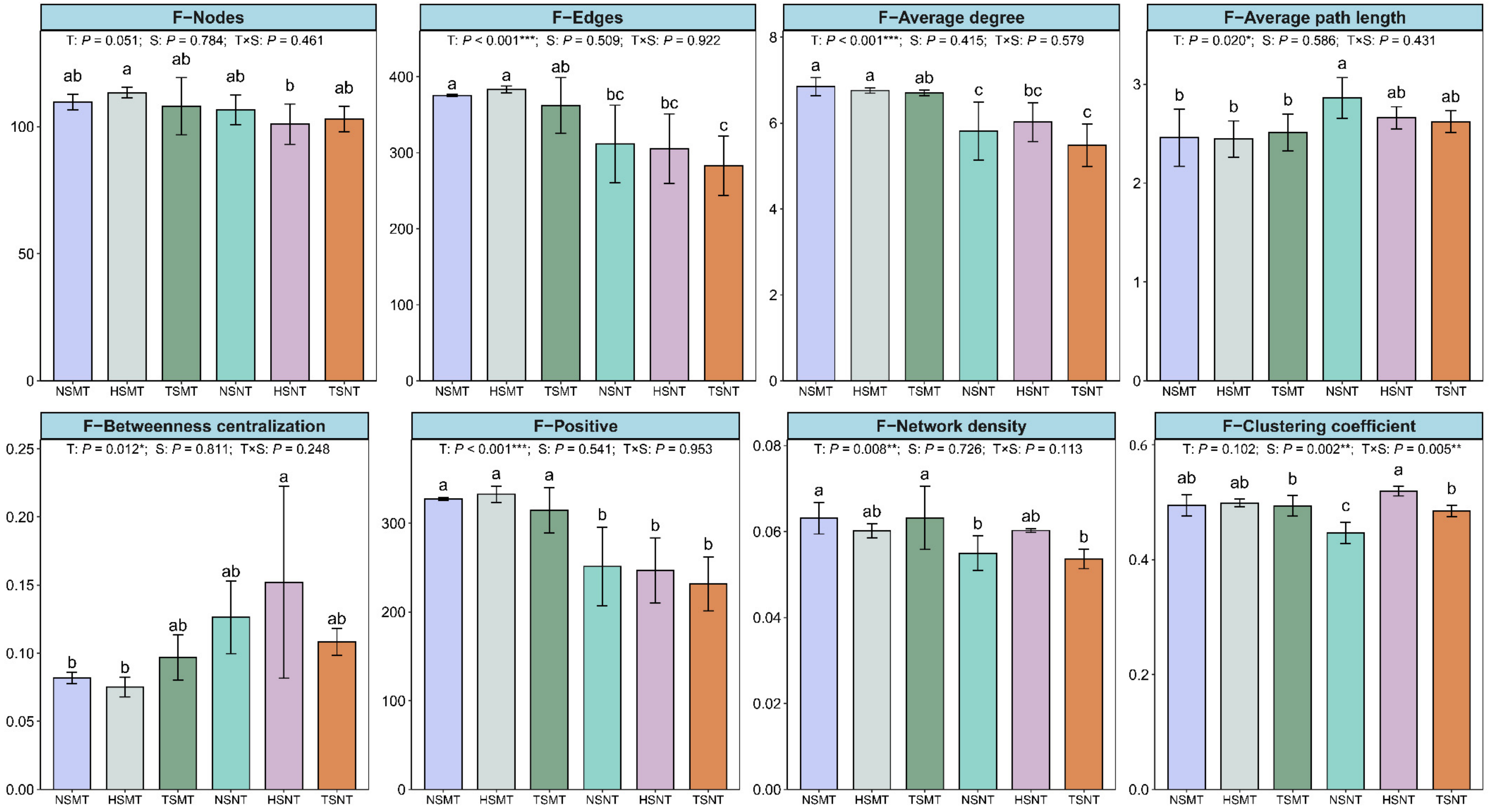

Figure S10. Topological properties of fungal co-occurrence networks. Different lowercase letters above boxplots indicate significant differences among treatments ($p < 0.05$).

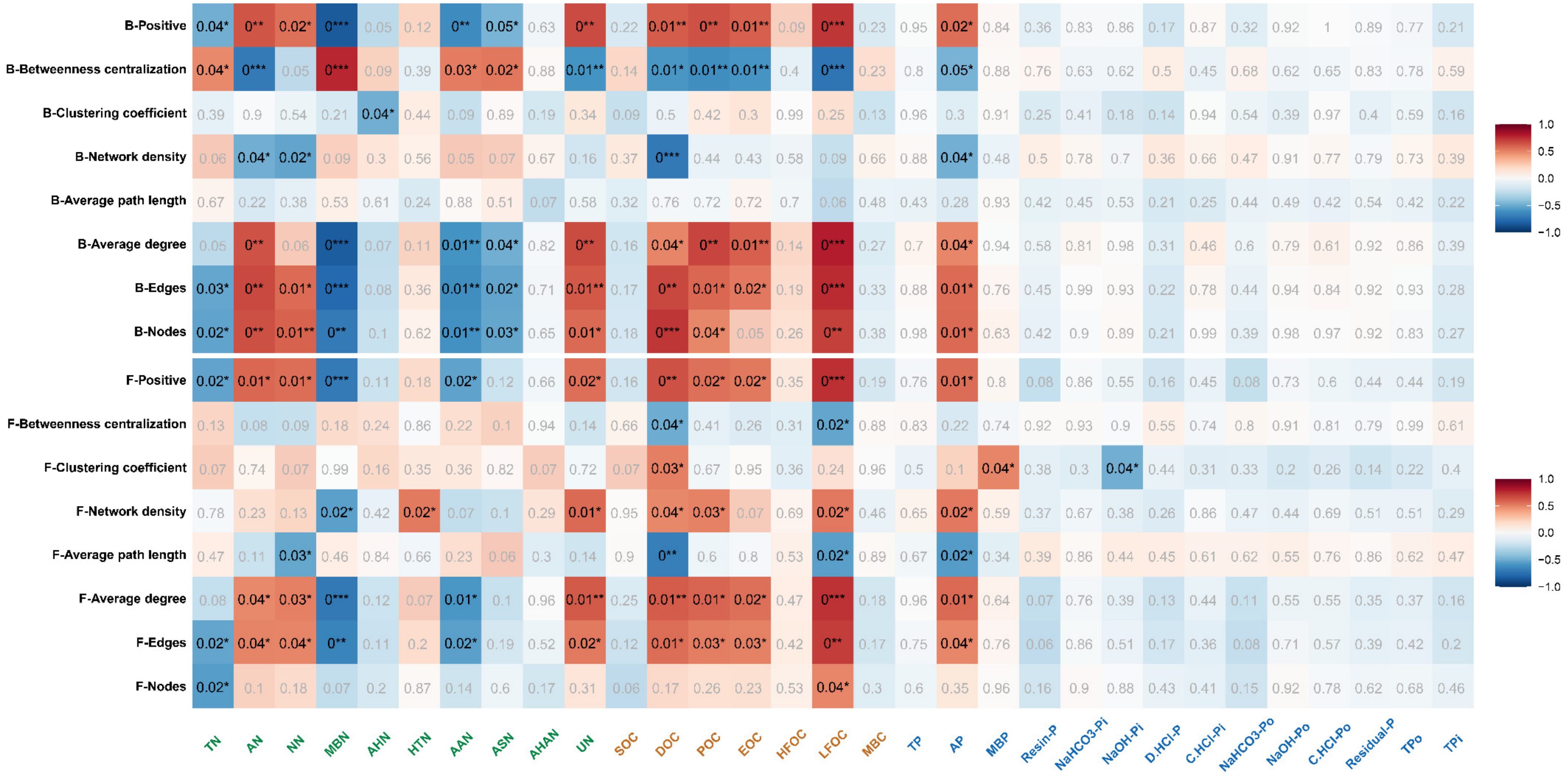

Figure S11. Correlation heatmap between topological properties of bacterial and fungal networks and soil carbon, nitrogen, and phosphorus fractions.

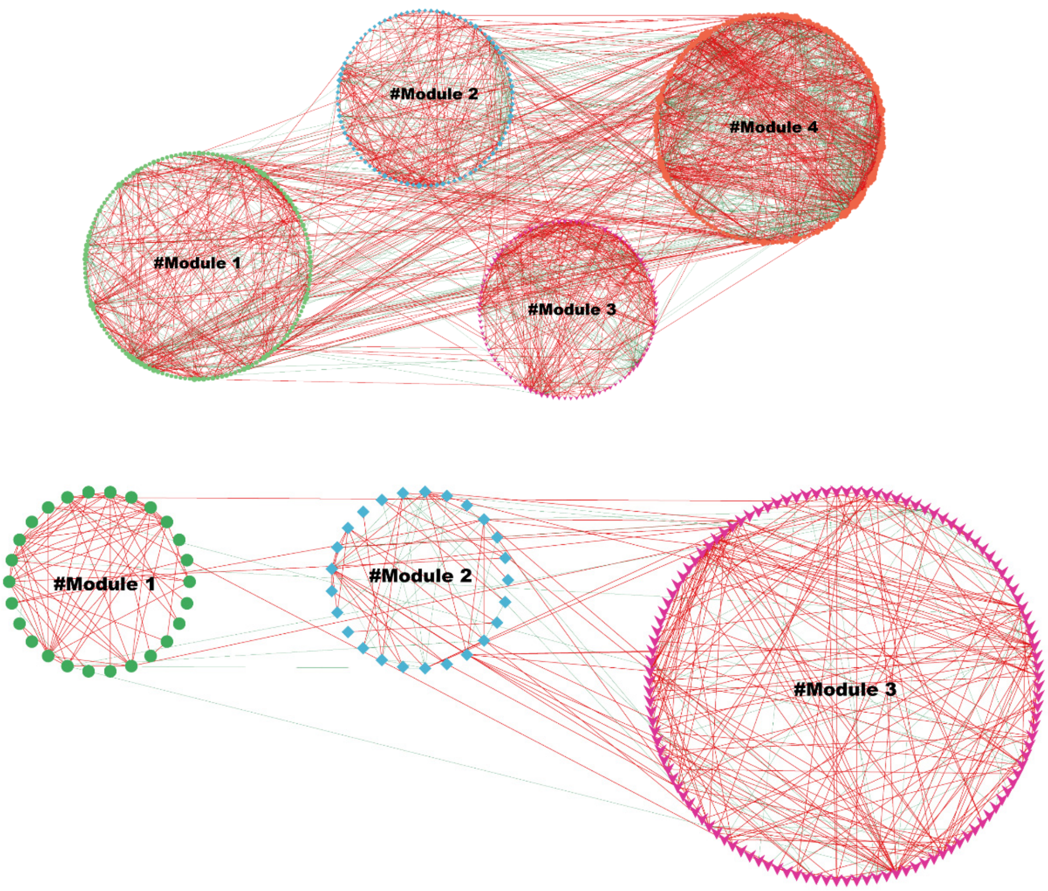

Figure S12. (A) Modular structure of the bacterial community co-occurrence network. The network was partitioned into four modules (labeled 1 to 4), containing 153, 92, 94, and 1155 microbial taxa at the genus level, respectively; (B) Modular structure of the fungal community co-occurrence network. The network was partitioned into three modules (labeled 1 to 3), containing 26, 25, and 121 microbial taxa at the genus level, respectively.

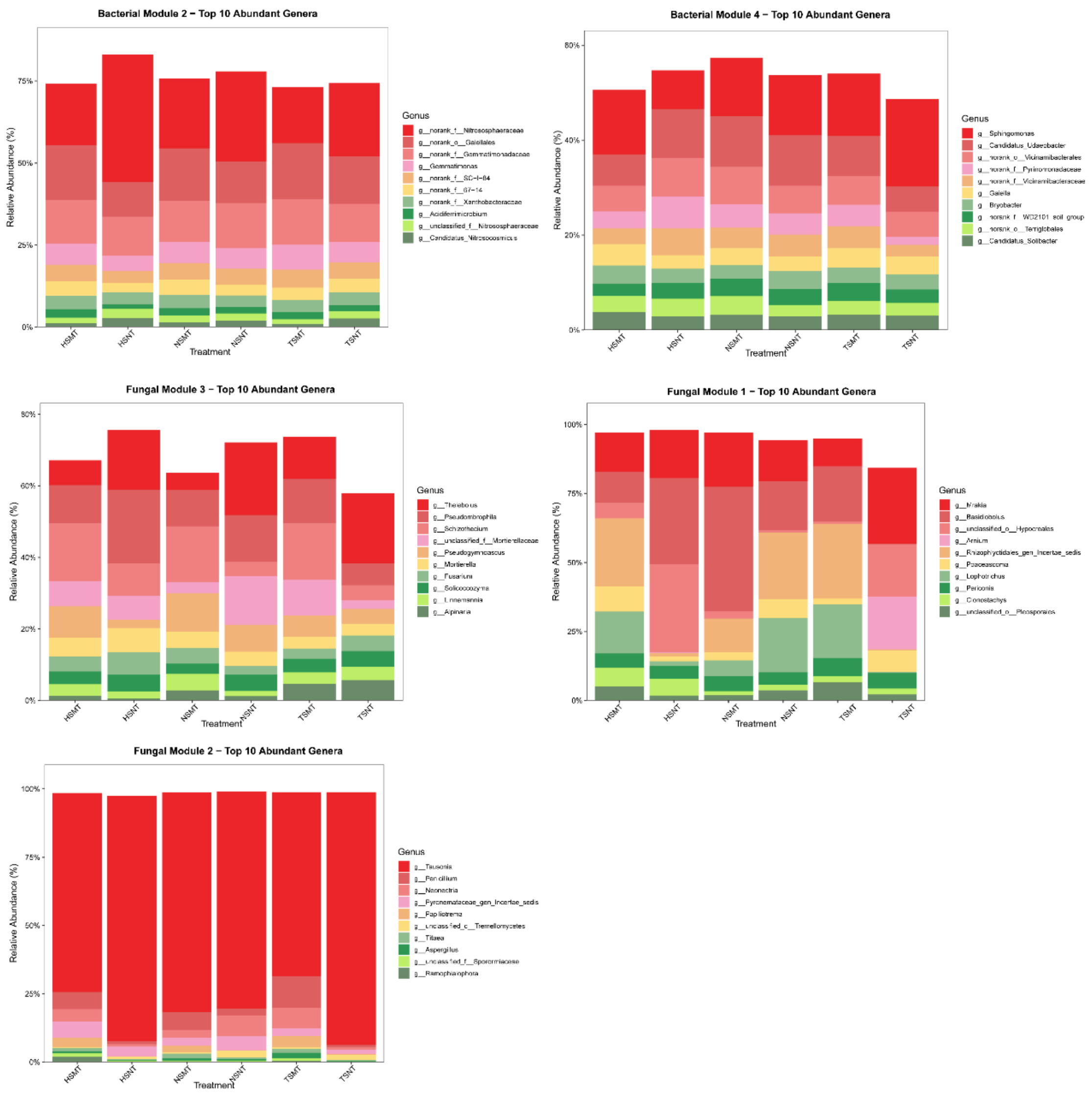

Figure S13. Top 10 genera ranked by microbial abundance within bacterial and fungal modules.

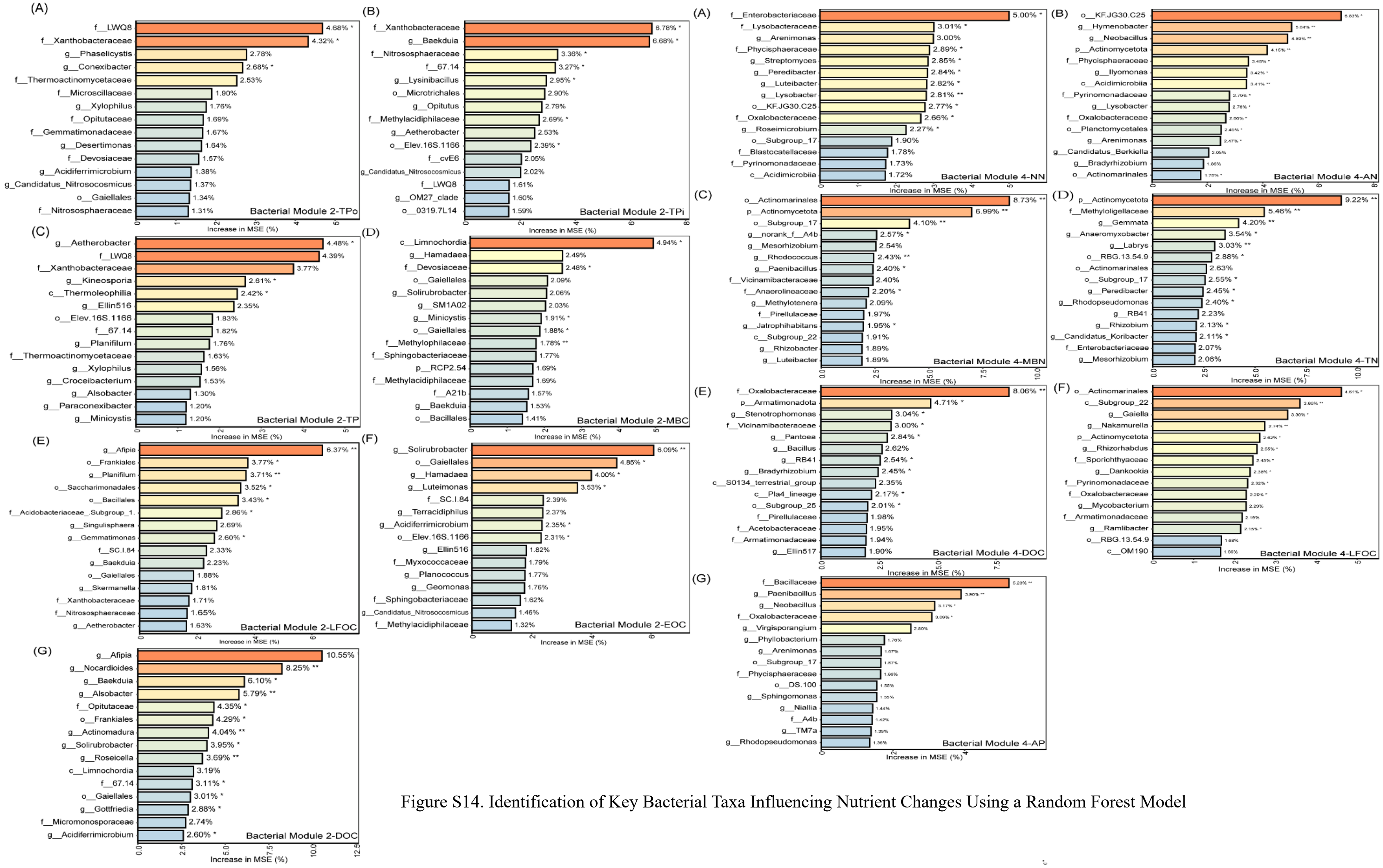

Figure S14. Identification of Key Bacterial Taxa Influencing Nutrient Changes Using a Random Forest Model

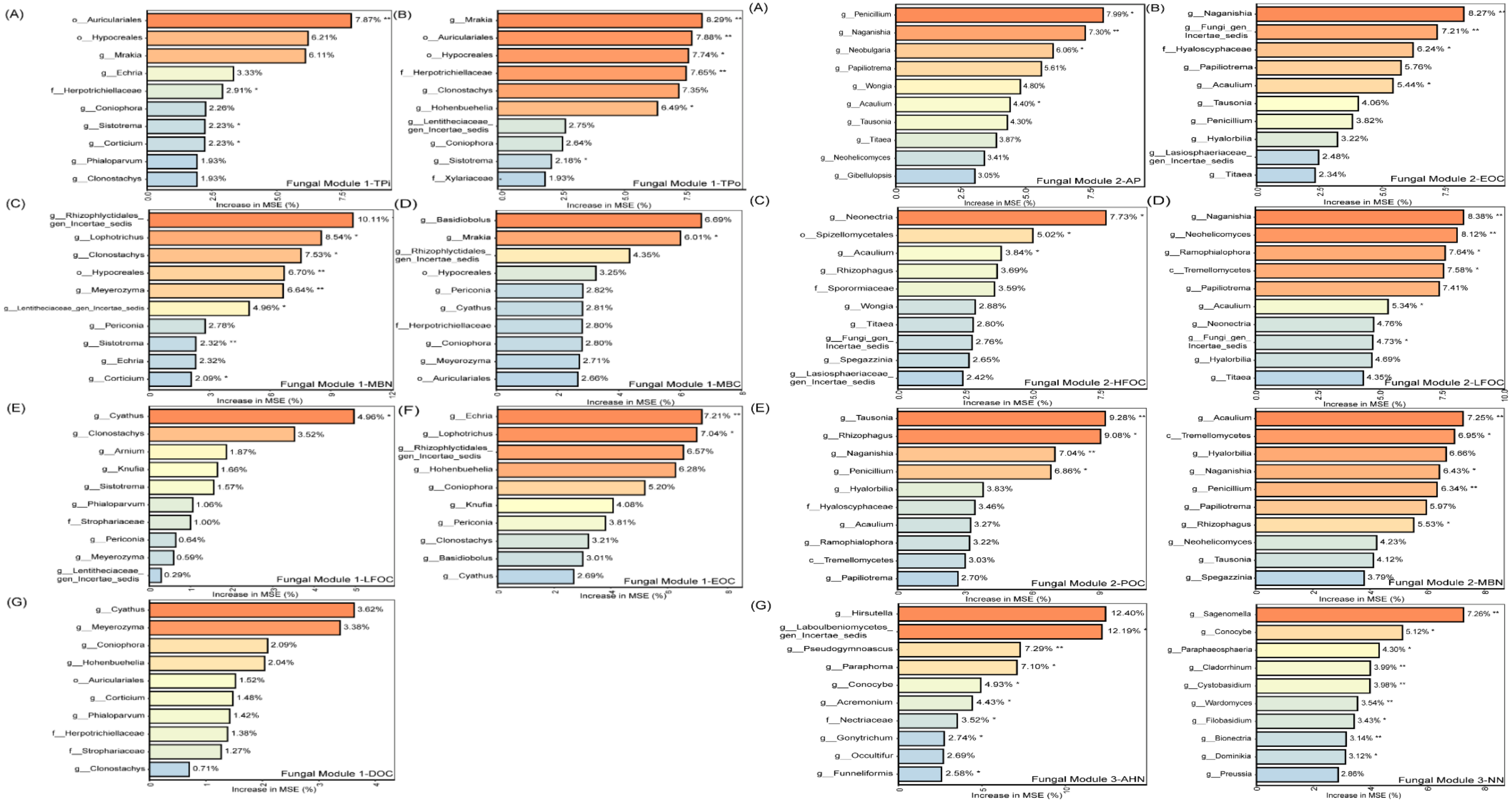

Figure S15. Identification of Key Fungal Taxa Influencing Nutrient Changes Using a Random Forest Model

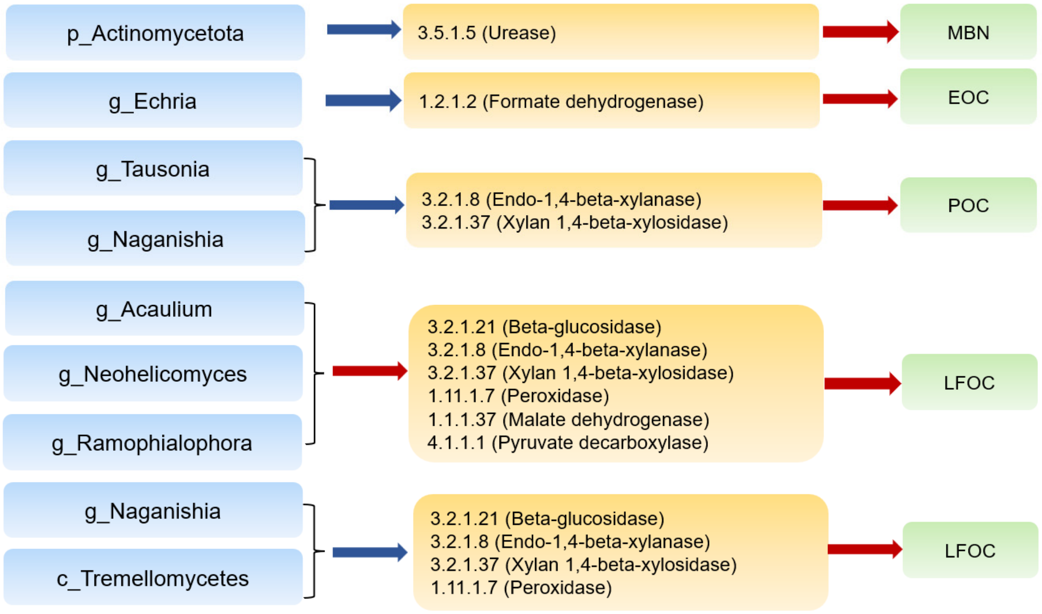

Figure S16. Pathway analysis of how core microorganisms mediate nutrient cycling by regulating the expression of functional enzyme genes. Red indicates positive regulation, and blue indicates negative regulation.

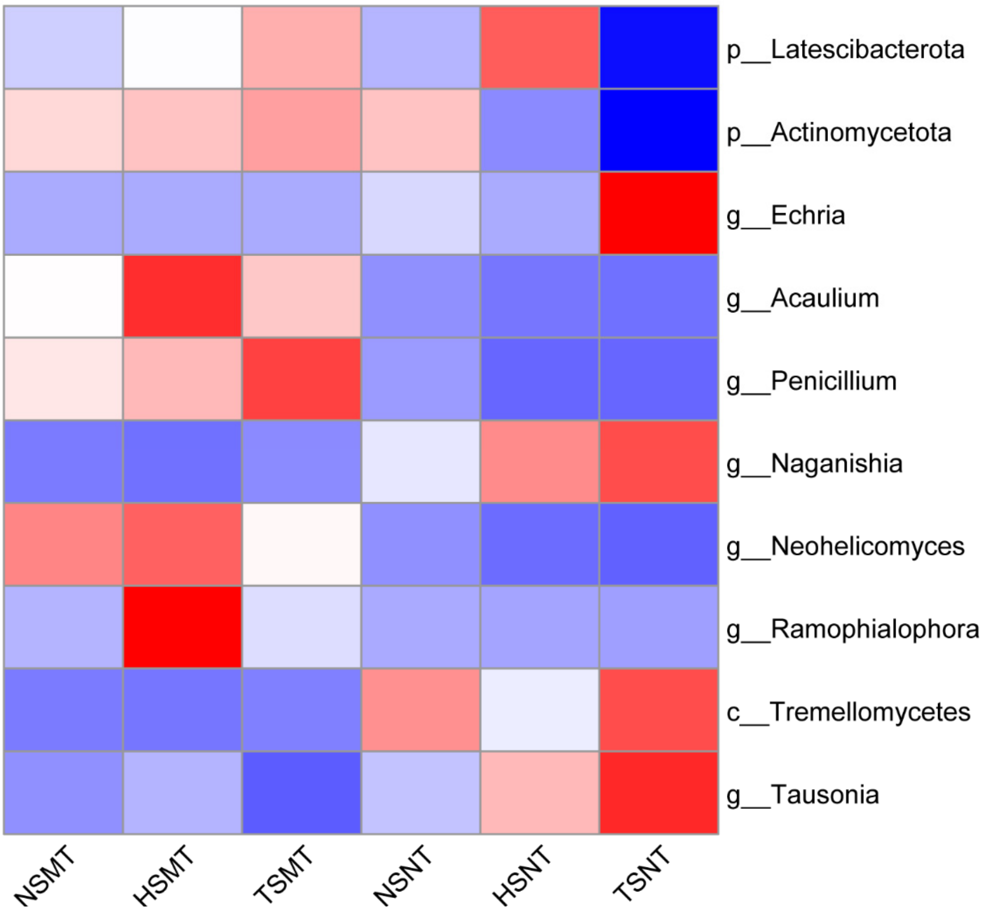

Figure S17 Abundance of Core Soil Microorganisms and Analysis of Variance under Different Tillage Practices
